# Vitamin D supplementation to prevent acute respiratory infections: systematic review and meta-analysis of stratified aggregate data

**DOI:** 10.1016/S2213-8587(24)00348-6

**Published:** 2025-02-21

**Authors:** David A Jolliffe, Carlos A Camargo, John D Sluyter, Mary Aglipay, John F Aloia, Peter Bergman, Heike A Bischoff-Ferrari, Arturo Borzutzky, Vadim Y Bubes, Camilla T Damsgaard, Francine M Ducharme, Gal Dubnov-Raz, Susanna Esposito, Davaasambuu Ganmaa, Clare Gilham, Adit A Ginde, Inbal Golan-Tripto, Emma C Goodall, Cameron C Grant, Christopher J Griffiths, Anna Maria Hibbs, Wim Janssens, Anuradha Vaman Khadilkar, Ilkka Laaksi, Margaret T Lee, Mark Loeb, Jonathon L Maguire, Paweł Majak, Semira Manaseki-Holland, JoAnn E Manson, David T Mauger, David R Murdoch, Akio Nakashima, Rachel E Neale, Hai Pham, Christine Rake, Judy R Rees, Jenni Rosendahl, Robert Scragg, Dheeraj Shah, Yoshiki Shimizu, Steve Simpson-Yap, Geeta Trilok Kumar, Mitsuyoshi Urashima, Adrian R Martineau

**Affiliations:** **Blizard Institute, Faculty of Medicine and Dentistry, Queen Mary University of London, London, UK**, (D A Jolliffe PhD, Prof A R Martineau PhD, Prof C J Griffiths DPhil)**; Centre for Applied Respiratory Research Innovation and Implementation, Wolfson Institute of Population Health, Queen Mary University of London, London, UK** (D A Jolliffe, Prof A R Martineau, Prof C J Griffiths)**; Department of Emergency Medicine, Massachusetts General Hospital, Harvard Medical School, Boston, MA, USA** (Prof C A Camargo Jr MD)**; School of Population Health, University of Auckland, Auckland, New Zealand** (J D Sluyter PhD, Prof R Scragg MD)**; Department of Pediatrics, St Michael’s Hospital, Toronto, ON, Canada** (M Aglipay MSc, Prof J L Maguire MD)**; Bone Mineral Research Center, Winthrop University Hospital, Mineola, NY, USA** (Prof J F Aloia MD)**; Department of Nutrition, Harvard School of Public Health, Boston, MA, USA** (D Ganmaa PhD)**; Department of Laboratory Medicine, Karolinska Institutet, Stockholm, Sweden** (Prof P Bergman MD)**; Department of Aging Medicine and Aging Research, University of Zurich, Zurich, Switzerland** (Prof H A Bischoff-Ferrari MD)**; Department of Pediatric Infectious Diseases and Immunology, School of Medicine, Pontificia Universidad Católica de Chile, Santiago, Chile** (A Borzutzky MD)**; Department of Nutrition, Exercise and Sports, University of Copenhagen, Frederiksberg, Denmark** (C T Damsgaard PhD)**; Exercise, Lifestyle and Nutrition Clinic, Edmond and Lily Safra Children’s Hospital, Tel Hashomer, Israel** (Prof G Dubnov-Raz MD)**; Paediatric Clinic, Department of Medicine and Surgery, University of Parma, Parma, Italy** (Prof S Esposito MD)**; Department of Non-Communicable Disease Epidemiology, London School of Hygiene and Tropical Medicine, London, UK** (C Gilham MSc, C Rake MSc)**; Department of Emergency Medicine, University of Colorado School of Medicine, Aurora, CO, USA** (Prof A A Ginde MD)**; Saban Pediatric Medical Center, Soroka University Medical Center, Faculty of Health Sciences, Ben-Gurion University, Beer Sheva, Israel** (I Golan-Tripto MD)**; Department of Health Research Methods, Evidence and Impact, McMaster University, Hamilton, ON, Canada** (E C Goodall PhD)**; Department of Paediatrics: Child & Youth Health, Faculty of Medical and Health Sciences, University of Auckland, Auckland, New Zealand** (Prof C C Grant MD)**; Department of Pediatrics, Case Western Reserve University School of Medicine, Cleveland, OH, USA** (A M Hibbs MD)**; University Hospitals Rainbow Babies and Children’s Hospital, Cleveland, OH, USA** (A M Hibbs)**; Universitair ziekenhuis Leuven, Leuven, Belgium** (Prof W Janssens MD)**; Hirabai Cowasji Jehangir Medical Research Institute, Maharashtra, India** (A V Khadilkar MD)**; Faculty of Medicine and Health Technology, University of Tampere, Tampere, Finland** (I Laaksi MD)**; Division of Pediatric Hematology/Oncology/Stem Cell Transplantation, Columbia University Medical Center, New York, NY, USA** (M T Lee MD)**; Department of Pathology and Molecular Medicine, McMaster University, Hamilton, ON, Canada** (Prof M Loeb MD)**; Department of Pediatric Pulmonology, Medical University of Lodz, Korczak Pediatric Center, Lodz, Poland** (P Majak MD)**; Department of Statistics, The Pennsylvania State University, Hershey, PA, USA** (Prof D T Mauger PhD)**; Department of Public Health, Epidemiology and Biostatistics, Institute of Applied Health Sciences, College of Medical and Dental Sciences, University of Birmingham, Birmingham, UK** (S Manaseki-Holland PhD)**; Department of Pathology, University of Otago, Christchurch, New Zealand** (Prof D R Murdoch MD)**; Jikei University School of Medicine, Tokyo, Japan** (A Nakashima MD, Prof M Urashima MD)**; Population Health Department, QIMR Berghofer Medical Research Institute, Queensland, Australia** (H Pham MPH)**; Department of Epidemiology, Geisel School of Medicine at Dartmouth, Lebanon, NH, USA** (J R Rees BM BCh); **Children’s Hospital, Pediatric Research Centre, University of Helsinki and Helsinki University Hospital, Helsinki, Finland** (J Rosendahl MD)**; Department of Paediatrics, University College of Medical Sciences, Delhi, India** (Prof D Shah MD)**; FANCL Research Institute, FANCL Corporation, Yokohama, Japan** (Y Shimizu PhD)**; Florey Institute for Neuroscience and Mental Health, The University of Melbourne, Parkville, VIC, Australia** (S Simpson-Yap PhD)**; Trivedi School of Biosciences, Ashoka University, Sonepat, Haryana, India** (Prof R E Neale PhD, Prof G Trilok-Kumar PhD)**; Departments of Paediatrics and of Social and Preventive Medicine, University of Montréal, Canada** (F M Ducharme MD)**; Division of Preventive Medicine, Brigham and Women’s Hospital and Harvard Medical School, Boston, MA, USA** (V Y Bubes PhD, Prof J E Manson MD)**; Neuroepidemiology Unit, Melbourne School of Population & Global Health, The University of Melbourne, Carlton, VIC, Australia** (S Simpson-Yap)**; Menzies Institute for Medical Research, University of Tasmania, Hobart, TAS, Australia** (S Simpson-Yap)**; The Centre For Military Medicine, Finland** (I Laaksi)

## Abstract

**Background:**

A 2021 meta-analysis of 37 randomised controlled trials (RCTs) of vitamin D supplementation for prevention of acute respiratory infections (ARIs) revealed a statistically significant protective effect of the intervention (odds ratio [OR] 0·92 [95% CI 0·86 to 0·99]). Since then, six eligible RCTs have been completed, including one large trial (n=15 804). We aimed to re-examine the link between vitamin D supplementation and prevention of ARIs.

**Methods:**

Updated systematic review and meta-analysis of data from RCTs of vitamin D for ARI prevention using a random effects model. Subgroup analyses were done to determine whether effects of vitamin D on risk of ARI varied according to baseline 25-hydroxyvitamin D (25[OH]D) concentration, dosing regimen, or age. We searched MEDLINE, EMBASE, the Cochrane Central Register of Controlled Trials, Web of Science, and the ClinicalTrials.gov between May 1, 2020 (end-date of search of our previous meta-analysis) and April 30, 2024. No language restrictions were imposed. Double-blind RCTs supplementing vitamin D for any duration, with placebo or lower-dose vitamin D control, were eligible if approved by a Research Ethics Committee and if ARI incidence was collected prospectively and pre-specified as an efficacy outcome. Aggregate data, stratified by baseline 25(OH)D concentration and age, were obtained from study authors. The study was registered with PROSPERO (no. CRD42024527191).

**Findings:**

We identified six new RCTs (19 337 participants). Data were obtained for 16 085 (83·2%) participants in three new RCTs and combined with data from 48 488 participants in 43 RCTs identified in our previous meta-analysis. For the primary comparison of any vitamin D versus placebo, the intervention did not statistically significantly affect overall ARI risk (OR 0·94 [95% CI 0·88–1·00], p=0·057; 40 studies; 61 589 participants; *I*^2^=26·4%). Pre-specified subgroup analysis did not reveal evidence of effect modification by age, baseline vitamin D status, dosing frequency, or dose size. Vitamin D did not influence the proportion of participants experiencing at least one serious adverse event (OR 0·96 [95% CI 0·90–1·04]; 38 studies; *I*^2^=0·0%). A funnel plot showed left-sided asymmetry (p=0·0020, Egger’s test).

**Interpretation:**

This updated meta-analysis yielded a similar point estimate for the overall effect of vitamin D supplementation on ARI risk to that obtained previously, but the 95% CI for this effect estimate now includes 1·00, indicating no statistically significant protection.

**Funding:**

None.

## Introduction

Acute respiratory infections (ARIs) are typically defined as any infection of the respiratory tract with symptom duration up to 21 days. Their contribution to global morbidity and mortality, with consequent strain on health-care systems, remains an ongoing problem. Evidence indicating that vitamin D supplementation could reduce risk of ARI arises from laboratory studies which show that vitamin D metabolites support innate immune responses to respiratory viruses,^1^ together with observational studies reporting independent associations of low circulating levels of 25-hydroxyvitamin D (25[OH]D, the widely accepted biomarker of vitamin D status) and increased risk of ARI.^2,3^

Randomised controlled trials (RCTs) of vitamin D for the prevention of ARIs have produced heterogeneous results, with some showing protection, and others reporting null findings. We previously did a meta-analysis of aggregate data from 48 488 participants in 43 RCTs,^4–45^ and found a modest protective overall effect of vitamin D that was stronger in trials which gave vitamin D daily, with doses of 400–1000 IU/day, were up to 12 months in length, and that were conducted among participants aged 1–15 years at enrolment.^46^ Since the date of our previous literature search (on May 1, 2020), six RCTs with 19 337 participants fulfilling the same eligibility criteria have been completed. We aimed to use data from these recent studies for inclusion in an updated meta-analysis of stratified aggregate data (trial-level, stratified by baseline vitamin D status and age) to determine whether vitamin D reduced ARI risk overall, and to evaluate whether effects of vitamin D on ARI risk varied according to baseline 25(OH)D concentration, dosing regimen (frequency, dose size, and trial duration), or age at enrolment.

## Methods

### Search strategy and selection criteria

This was a systematic review and meta-analysis. Methods were pre-specified in a protocol that was registered with the PROSPERO International Prospective Register of Systematic Reviews.^46^ The study was registered with PROSPERO (no. CRD42024527191). Details of Research Ethics Committee approvals to conduct this study are included in the appendix (p 9).

Double-blind, randomised controlled trials of supplementation with vitamin D_3_, vitamin D_2,_ or 25(OH)D of any duration, with participants of any age and with a placebo or blinded lower-dose vitamin D control for the primary prevention of ARI, were eligible for inclusion if they had been approved by a Research Ethics Committee and if data on incidence of ARI were collected prospectively and pre-specified as an efficacy outcome. The latter requirement was imposed to minimise misclassification bias (prospectively designed instruments to capture ARI events were deemed more likely to be sensitive and specific for this outcome). Studies reporting results of long-term follow-up of primary RCTs were excluded.

Two investigators (ARM and DAJ) searched MEDLINE, EMBASE, the Cochrane Central Register of Controlled Trials (CENTRAL), Web of Science, and the ClinicalTrials.gov registry using the electronic search strategies described in the appendix (pp 4–6), for studies published since May 1, 2020. Searches were regularly updated up to, and including, April 30, 2024. No language restrictions were imposed. These searches were supplemented by searching review articles and reference lists of trial publications. Collaborators were asked if they knew of any additional eligible RCTs.

### Data analysis

Details of the data collection process are provided in the appendix (p 6). The primary outcome of the meta-analysis was the proportion of participants experiencing one or more ARI, with the definition of ARI encompassing events classified as upper respiratory tract infection (URI), lower respiratory tract infection (LRI), and ARI of unclassified location (ie, infection of the upper respiratory tract, lower respiratory tract, or both). Secondary outcomes were: incidence of URIs and LRIs, analysed separately; incidence of emergency department attendance or hospital admission for ARIs (or both); death due to ARIs or respiratory failure; use of antibiotics to treat an ARI; absence from work or school due to ARIs; incidence of serious adverse events; death due to any cause; and incidence of potential adverse reactions to vitamin D (hypercalcaemia and renal stones).

We used the Cochrane Collaboration Risk of Bias tool^47^ to assess the following variables: sequence generation, allocation concealment, blinding of participants, personnel and outcome assessors, completeness of outcome data, evidence of selective outcome reporting, and other potential threats to validity. Study quality was assessed independently by two investigators (ARM and DAJ), except for the six trials for which DAJ or ARM were investigators, which were assessed by CAC and JDS. Discrepancies were resolved by consensus.

Data were analysed by DAJ; results were checked and verified by JDS. Our meta-analysis approach followed published guidelines.^48^ The primary comparison was of participants randomised to any vitamin D supplement versus placebo; this was performed for all of the outcomes listed above. For trials that included higher-dose, lower-dose, and placebo groups, data from higher-dose and lower-dose arms were pooled for analysis of the primary comparison. A secondary comparison of participants randomly assigned to higher versus lower doses of vitamin D was performed for the primary outcome only.

The log odds ratio and its standard error were calculated for each outcome within each trial from the proportion of participants experiencing one or more events in the intervention versus the control group. Odds ratios were pre-specified as the effects measure in all analyses in our study protocol, in order to avoid potential pitfalls when using risk ratios in meta-analyses.^49^ This approach is entirely in accordance with the Cochrane Handbook’s guidelines.^50^ It also allows readers to make a direct comparison of results from the current analysis with those of our previous meta-analyses, which also used this methodology.^46,51–53^ Where trials reported zero events in a given group, Haldane correction was applied.^54^ For trials where randomisation was stratified by study site, proportions were corrected for clustering using published methods.^55^ Proportions (events/group size) were then meta-analysed in a random-effects model using the Metan package^56^ within STATA IC version 14.2 to obtain an overall odds ratio (OR) with a 95% CI and a measure of heterogeneity summarised by the *I*^2^ statistic and its corresponding p value.

To explore reasons for heterogeneity of effect of the intervention between trials we performed a stratified analysis according to baseline vitamin D status (serum 25[OH]D <25 *vs* 25–49·9 *vs* 50–74·9 *vs* ≥75 nmol/L) and according to age at baseline (<1 *vs* 1–15 *vs* 16–64 *vs* ≥65 years). We also conducted subgroup analyses according to vitamin D dosing regimen (administration of daily *vs* weekly *vs* monthly or less frequent doses), dose size (daily equivalent <400 IU *vs* 400–1000 IU *vs* 1001–2000 IU *vs* >2000 IU), trial duration (≤12 months *vs* >12 months), and presence of airway disease (trial restricted to participants with asthma *vs* those restricted to participants with chronic obstructive pulmonary disease [COPD] *vs* those in which participants without airway disease were eligible). The thresholds for baseline 25(OH)D concentration used in subgroup analyses were selected a priori on the basis that they represent cutoffs that are commonly used to distinguish profound vitamin D deficiency (<25 nmol/L), moderate vitamin D deficiency (25–49·9 nmol/L), and potentially sub-optimal vitamin D status (50–74·9 nmol/L).^57^

To investigate factors associated with heterogeneity of the effect between statistically significant (alpha 5%) subgroups of trials, we performed multivariable meta-regression analysis on trial-level characteristics, the full details of which are described in the appendix (p 10).

For the primary analysis, the likelihood of publication bias was investigated through the construction of a contour-enhanced funnel plot.^58^ We used the five Grading of Recommendations, Assessment, Development, and Evaluation considerations (study limitations, consistency of effect, imprecision, indirectness, and publication bias)^59^ to assess the quality of the body of evidence contributing to analyses of the primary efficacy outcome and major secondary outcomes of our meta-analysis.

We conducted two exploratory sensitivity analyses for the primary comparison of the primary outcome: one excluded RCTs where risk of bias was assessed as being unclear, and the other excluded RCTs in which incidence of ARI was not the primary or co-primary outcome.

Due to the relatively low level of heterogeneity between trials entering into the primary outcome model, we also estimated the overall primary outcome using a fixed effects model. Additionally, where five trials or less contributed data to a subgroup analysis, we also estimated effects using the Hartung–Knapp–Sidik–Jonkman model (appendix p 17).^60^

## Results

**Figure 1:**
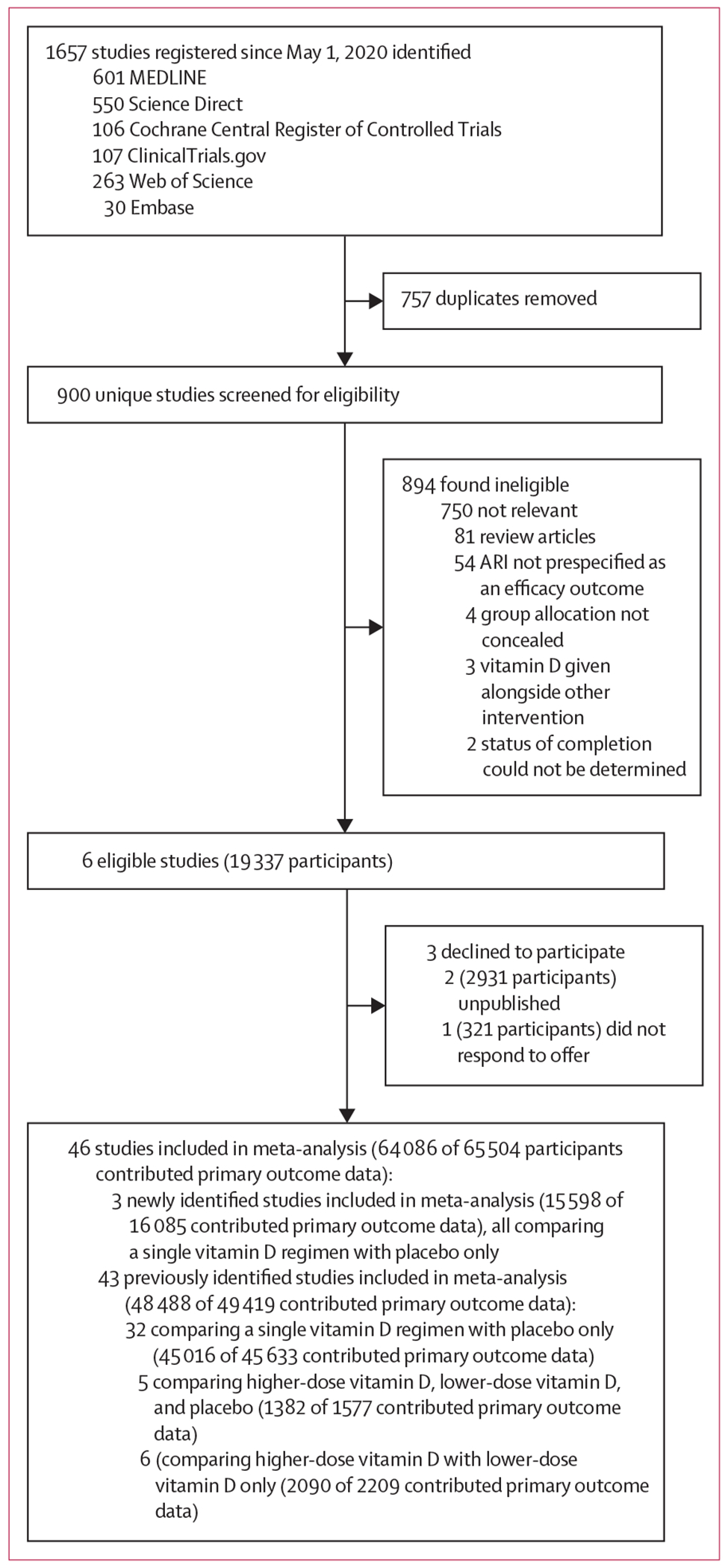
Study selection. ARI=acute respiratory infection.

Our updated search (studies published from May 1, 2020 to April 30, 2024) identified a total of 900 studies that were assessed for eligibility, of which six studies with a total of 19 337 randomly assigned participants met the eligibility criteria. Studies for which the full text was reviewed before exclusion due to ineligibility are listed in the appendix (p 11). All six of the identified eligible studies compared effects of a single vitamin D regimen versus placebo only. Data for the primary outcome (proportion of participants with one or more ARI) were obtained for 15 598 (97·0%) of 16 085 participants in three studies^13,61,62^ and were added to our database of 43 previously identified eligible studies (described elsewhere),^46^ bringing the total number of participants contributing data to the analysis of our primary outcome to 64 086 (97·8%) of 65 504 participants from 46 studies (figure 1).

Trials were conducted in 24 different countries on five continents, and enrolled male and female participants from birth to 100 years of age^4–38,40–45,61–64^ (table 1). Baseline serum 25(OH)D concentrations were determined in 38 of 46 trials: mean baseline 25(OH)D concentration ranged from 18·9 nmol/L to 90·9 nmol/L (to convert to ng/mL, divide by 2·496). 45 studies administered oral vitamin D_3_ to participants in the intervention group, and one study administered oral 25(OH)D. Vitamin D was given as monthly to 3-monthly bolus doses in 13 studies; as weekly doses in seven studies; as daily doses in 24 studies; and as a combination of bolus and daily doses in two studies. Trial duration ranged from 7 weeks to 5 years. Incidence of ARI was a primary or co-primary outcome for 25 studies, and a secondary outcome for 21 studies.

Details of the risk of bias assessment are provided in the appendix (p 12). Five trials were assessed as being at unclear risk of bias due to high loss to follow-up. In the trial by Laaksi and colleagues,^22^ 37% of randomly assigned participants were lost to follow-up. In the trial by Dubnov-Raz and colleagues,^12^ 52% of participants did not complete all symptom questionnaires. In the unpublished trial by Reyes and colleagues, loss to follow-up ranged from 33% to 37% across the three study groups,^63^ and in the unpublished trial by Golan-Tripto and colleagues,^64^ 50% of participants were lost to follow-up. Finally, in the trial by Huang and colleagues,^61^ we detected uncertainty around blinding of outcome assessment within the study team, uncertainty around methodology for dealing with incomplete data, and selective outcome reporting, which we were unable to resolve with the authors. All other trials were assessed as being at low risk of bias for all seven aspects assessed.

**Figure 2:**
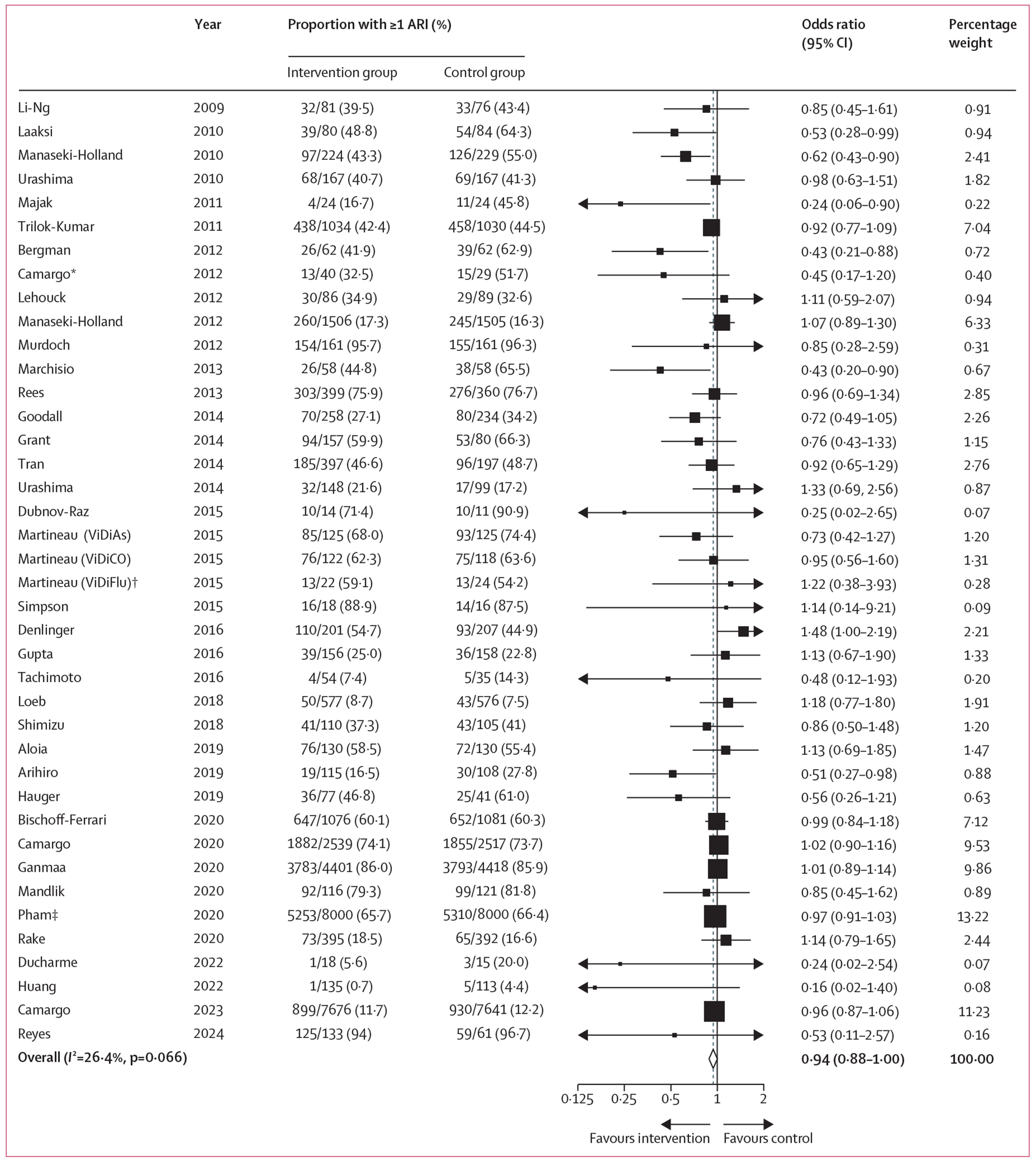
Forest plot of placebo-controlled RCTs reporting proportion of participants experiencing one or more acute respiratory infection Weights are from random effects analysis. The numerator is the number of participants who reported an ARI on at least one survey. The ARI outcomes for participants who completed fewer than five surveys and who did not report an ARI (N=2239; 14%) were estimated based on the percent affected among those who completed all five surveys (N=12 152; 76%). ARI=acute respiratory infection. RCT=randomised controlled trial. *Proportions for this trial were corrected for cluster randomisation using the calculated design effect of 3·49. †This analysis includes data from the subset of ViDiFlu trial participants who were randomised to vitamin D versus placebo control; correction for cluster randomisation was not possible due to the lack of power. ‡For this trial, participants were asked to report the occurrence of ARI during the one month prior to completing each annual survey (max surveys=5).

For the primary comparison of any vitamin D supplement versus placebo control, supplementation did not result in a statistically significant reduction in the proportion of participants experiencing at least one ARI (OR 0·94 [95% CI 0·88–1·00], p=0·057; 61 589 participants in 40 studies; figure 2, table 2; appendix p 16). Between-trial heterogeneity was modest: *I*^2^=26·4% (p for heterogeneity 0·07).

For the secondary comparison of higher-dose versus lower-dose vitamin D, we observed no statistically significant difference in the proportion of participants with at least one ARI (OR 0·87 [95% CI 0·73–1·04]; 3047 participants in 11 studies; *I*^2^=0·0%, p for heterogeneity 0·50; appendix p 19).

To investigate reasons for the observed heterogeneity of effect for the primary comparison of any vitamin D supplement versus placebo control, we stratified this analysis by two participant-level factors (baseline vitamin D status and age) and by four trial-level factors (dose frequency, dose size, trial duration, and airway disease comorbidity). No statistically significant effect of vitamin D was seen for participants with baseline 25(OH)D less than 25 nmol/L (OR 0·98 [95% CI 0·80–1·20]; 3806 participants in 22 studies), 25–49·9 nmol/L (1·03 [0·94–1·13]; 11 618 participants in 31 studies), 50–74·9 nmol/L (0·90 [0·80–1·02]; 11 214 participants in 32 studies), or 75 nmol/L or greater (0·97 [0·87–1·07]; 11 815 participants in 28 studies; table 2, appendix p 20). A statistically significant protective effect of vitamin D was seen for participants aged 1–15 years (OR 0·74 [95% CI 0·60–0·92]; 11 944 participants in 16 studies), but not in participants aged <1 year (0·95 [0·82–1·10]; 5697 participants in five studies), 16–64 years (0·95 [0·86–1·05]; 14 498 participants in 23 studies), or 65 years or older (0·97 [0·92–1·02]; 29 583 participants in 18 studies; table 2, appendix p 24). With regard to dosing frequency, a statistically significant protective effect was seen for trials where vitamin D was given daily (OR 0·84 [95% CI 0·73–0·97]; 21 552 participants in 21 studies), but not for trials in which it was given weekly (0·97 [0·88–1·06]; 12 789 participants in seven studies), or monthly to 3-monthly (0·98 [0·93–1·03]; 27 248 participants in 12 studies; table 2, appendix p 21). Statistically significant protective effects of the intervention were also seen in trials where vitamin D was administered at daily equivalent doses of 400–1000 IU (OR 0·70 [95% CI 0·55–0·89]; 2305 participants in ten studies), but not where the daily dose equivalent was less than 400 IU (0·76 [0·41–1·41]; 2133 participants in two studies), 1001–2000 IU (0·97 [0·92–1·01]; 49 457 participants in 19 studies), or greater than 2000 IU (1·05 [0·84–1·31]; 6906 participants in seven studies; table 2, appendix p 22). Statistically significant protective effects were also seen for trials with a duration of 12 months or less (OR 0·85 [95% CI 0·76–0·95]; 24 678 participants in 32 studies) but not in those lasting more than 12 months (0·99 [0·95–1·04]; 36 911 participants in eight studies; table 2, appendix p 23).

Statistically significant protective effects of vitamin D were not seen in trials that exclusively enrolled participants with asthma, or trials that exclusively enrolled participants with COPD, or trials in which participants without airway disease were eligible (table 2, appendix p 25).

Multivariable meta-regression analysis of trial-level subgroups did not identify any statistically significant interactions (p values for interaction <0·05) between allocation to vitamin D versus placebo and dose frequency, dose size, trial duration, or participant age (appendix p 17).

Meta-analysis of secondary outcomes was performed for results of placebo-controlled trials only (ie, not for RCTs that compared higher-dose *vs* lower-dose vitamin D; table 3). Overall, without consideration of participant-level or trial-level factors, vitamin D supplementation did not have a statistically significant effect on the proportion of participants with one or more URI, LRI, hospitalisations or emergency department attendances for ARIs, death due to ARIs or respiratory failure, courses of antimicrobials for an ARI, work or school absences due to ARIs, serious adverse events of any cause, death due to any cause, or episodes of hypercalcaemia or renal stones.

A funnel plot for the proportion of participants experiencing at least one ARI (appendix p 26) showed left-sided asymmetry, confirmed with an Egger’s regression test^65^ (p=0·0020). This might reflect heterogeneity of effect across trials, or publication bias arising from omission of small trials showing non-protective effects of vitamin D from the meta-analysis.^66^ Given the latter possibility, the quality of the body of evidence contributing to analyses of the primary efficacy outcome and major secondary outcomes was downgraded to moderate (appendix p 15).

Results of exploratory sensitivity analyses are presented in the appendix (p 16). Meta-analysis of the proportion of participants in placebo-controlled trials experiencing at least one ARI, excluding four studies assessed as being at unclear risk of bias,^12,22,61,63^ did not reveal a statistically significant protective effect of any vitamin D supplementation (OR 0·95 [95% CI 0·90–1·01]; 60 958 participants in 36 studies), consistent with the main analysis. Similarly, sensitivity analyses for the same outcome, one excluding 19 placebo-controlled trials that investigated ARI as a secondary outcome, and another excluding three placebo-controlled trials designed to detect an effect of vitamin D on recurrent ARI,^18,29,31^ did not show a statistically significant protective effect (0·90 [0·79–1·02]; 9975 participants in 21 studies, and 0·96 [0·91–1·02]; 60 706 participants in 37 trials, respectively).

Due to the relatively low level of between-trial heterogeneity (*I*^2^=26·4%), we analysed the primary outcome using a fixed effects model, which yielded a very similar effect estimate (OR 0·96 [95% CI 0·93–1·00]; p=0·047).

## Discussion

This update to our 2021 meta-analysis of RCTs of vitamin D supplementation for the prevention of ARI includes new primary outcome data from an additional 15 598 participants in three studies completed since May, 2020, bringing the total number of participants contributing data to 64 086 from 46 trials. The point estimate of the overall effect of vitamin D supplementation on ARI risk obtained in the current analysis (0·94) is similar to that yielded by our previous meta-analysis (0·92). However, in contrast to our previous work,^46^ the 95% CI for this effect now spans 1·00. Although statistically significant protection was seen within some trial subsets (daily dosing trials, trials that administered 400–1000 IU/day, trials conducted for 12 months or less, and trials in participants aged 1–15 years), meta-regression analysis did not yield evidence to suggest that effects of vitamin D were modified by any of these factors.

Heterogeneity of results from the current meta-analysis is somewhat lower than that obtained from our previous meta-analysis (*I*^2^=26·4% in the current analysis *vs* 35·6% previously). This difference suggests that greater confidence can be placed in the findings of the current analysis versus our previous one. It is possible that the previous overall finding of a protective effect of any vitamin D supplement was driven by small study effects, as evidenced by left-sided asymmetry shown in the funnel plot (appendix p 24).^66^

The current study has several strengths. It contains the latest aggregate RCT data available worldwide, including stratified data for subgroups of baseline vitamin D status and age, and new data from a very large trial (n=15 804).^62^ The larger sample size provides improved statistical power to perform subgroup analyses and interrogate heterogeneity of effects across trials. Nevertheless, formal demonstration of effect modification is challenging and will likely require even larger sample sizes.

Our work also has limitations. Some trials did not respond to our invitation to contribute data for meta-analysis (figure 1 and appendix p 11), at least one of which reported protective effects of vitamin D against ARI,^67^ therefore potentially biasing our results towards the null. We meta-analysed aggregate (trial-level) data, rather than individual participant data. However, we did contact authors to get unpublished estimates of effect that were stratified by pre-defined baseline 25(OH)D levels and age, harmonised across studies, thus, we were able to obtain accurate data for the major participant-level potential effect-modifiers of interest. As with our previous update to the meta-analysis of this research question, there are still relatively few RCTs that have compared effects of lower-dose versus higher-dose vitamin D. Paucity of data in this area limited our power for this secondary comparison. We lacked the data to investigate race or ethnicity and obesity as potential effect-modifiers. We also could not account for other factors that might influence the efficacy of vitamin D supplements for ARI prevention (eg, taking the supplement with or without food, calcium intake, and vitamin A status) or secular trends that might influence trial findings, such as the increased societal use of vitamin D supplements.^68^ Concurrent use of supplements containing vitamin D by participants randomly assigned to the control group would effectively render these as higher-dose versus lower-dose trials and potentially drive results toward the null. Another potential limitation is illustrated by the funnel plot, which suggests that the overall effect size might have been over-estimated due to publication bias; we have attempted to mitigate this problem by inclusion of data from unpublished studies identified by searching ClinicalTrials.gov where this was obtainable. Finally, sparse-data bias^69^ could have affected the overall effect estimates and between-study heterogeneity estimates of subgroup analyses where one category included data from five or fewer trials.

In summary, this updated meta-analysis of data from RCTs of any vitamin D supplementation for the prevention of ARI yielded a similar point estimate for the overall effect of vitamin D supplementation on ARI risk to that obtained previously, but the 95% CI for this effect now includes 1·00, indicating no statistically significant protection.

## Supplementary Material

Supplementary

## Figures and Tables

**Table 1:** Characteristics of the trials and their participants

	Participants (Male:Female)	Mean age, years (SD) [range]	25(OH)D assay, EQA scheme	Mean baseline 25(OH)D, nmol/L (SD)	Baseline 25(OH)D <25 nmol/L (%)	Mean attained 25(OH)D, intervention group, nmol/L (SD)	Intervention: Control (total)	Oral dose of vitamin D_3_, intervention group	Control	Trial duration	ARI definition	n contributing data/n randomised (%)
**ARI primary outcome**												
Li-Ng 2009;^25^ USA	Healthy adults (34:128)	57·9 (13·6) [21·4–80·6]	RIA (DiaSorin), DEQAS	63·7 (25·5)	3/150 (2·0%)	88·5 (23·2)	84:78 (162)	50 μg daily	Placebo	3 months	URI: ≥2 URI symptoms in absence of allergy symptoms	157/162 (96·9%)
Urashima 2010;^45^ Japan	School children (242:188)	10·2 (2·3) [6·0–15·0]	Not determined	Not determined	Not determined	Not determined	217:213 (430)	30 μg daily	Placebo	4 months	URI: influenza A/B diagnosed by RIDT or RIDT-negative ILI	334/430 (77·7%)
Laaksi 2010;^22^ Finland	Military conscripts (164:0)	19·1 (0·6) [18·0–21·0]	EIA (IDS OCTEIA)	75·9 (18·7)	0/73 (0·0%)	71·6 (22·9)	80:84 (164)	10 μg daily	Placebo	6 months	ARI: medical record diagnosis	164/164 (100%)
Manaseki-Holland 2012;^28^ Afghanistan	Infants (1591:1455)	0·5 (0·3) [0·0–1·0]	..	Not determined	Not determined	32·7 (17·1)	1524:1522 (3046)	2·5 mg bolus 3-monthly	Placebo	1·5 years	LRI: pneumonia confirmed by chest radiograph	3011/3046 (98·9%)
Murdoch 2012;^35^ New Zealand	Healthy adults (81:241)	48·1 (9·7) [18·0–67·6]	LC-MS/MS, DEQAS	72·1 (22·1)	5/322 (1·6%)	123·6 (27·5)	161:161 (322)	2 × 5 mg bolus monthly then 2·5 mg bolus monthly	Placebo	1·5 years	URI: assessed with symptom score	322/322 (100%)
Marchisio 2013;^31^ Italy	Children with recurrent acute otitis media (64:52)	2·8 (1·0) [1·3–4·8]	CLA (DiaSorin), ISO9001	65·3 (17·3)	2/116 (1·7%)	90·3 (21·1)	58:58 (116)	25 μg daily	Placebo	6 months	URI: doctor-diagnosed acute otitis media	116/116 (100%)
Goodall 2014;^16^ Canada	Healthy university students (218:382)	19·6 (2·2) [17·0–33·0]	Not determined	Not determined	Not determined	Not determined	300:300 (600)	0·25 mg weekly (2 × 2 factorial with gargling)	Placebo	8 weeks	URI: self-reported cold	492/600 (82·0%)
Urashima 2014;^44^ Japan	High school students (162:85)	16·5 (1·0) [15·0–18·0]	Not determined	Not determined	Not determined	Not determined	148:99 (247)	50 μg daily	Placebo	2 months	URI: influenza A diagnosed by RIDT or RIDT-negative ILI	247/247 (100%)
Simpson 2015;^41^ Australia	Healthy adults (14:20)	32·2 (12·2) [18·0–52·0]	LC-MS/MS, DEQAS	67·9 (23·0)	0/33 (0·0%)	Not determined	18:16 (34)	0·5 mg weekly	Placebo	17 weeks	ARI assessed with symptom score	34/34 (100%)
Dubnov-Raz 2015;^12^ Israel	Adolescent swimmers with vitamin D insufficiency (34:20)	15·2 (1·6) [12·9–18·6]	RIA (DiaSorin), DEQAS	60·4 (11·9)	0/54 (0·0%)	73·7 (16·6)	27:27 (54)	50 μg daily	Placebo	12 weeks	URI assessed with symptom score	25/54 (46·3%)
Ginde, 2016;^15^ USA	Institutionalised older adults (45:62)	80·7 (9·9) [60·0–95·0]	LC-MS/MS, VDSP	57·3 (22·7)	12/107 (11·2%)	Not determined	55:52 (107)	2·5 mg bolus monthly + ≤25 μg per day equivalent	Placebo + 10–25 μg per day equivalent	1 year	ARI: medical record diagnosis	107/107 (100%)
Aglipay 2017;^4^ Canada	Healthy children (404:296)	2·7 (1·5) [1·0–5·0]	CLA (Roche ELECSYS)	90·9 (20·9)	1/703 (0·1%)	High dose: 121·6 (2·2); Low dose: 91·9 (1·7)	349:354	50 μg daily	10 μg daily	4–8 months (mean 6·3 months)	URI: lab confirmed	699/703 (99·4%)
Arihiro 2019;^6^ Japan	Adults with diagnosis of inflammatory bowel disease (146:91)	44·5 (13·2) [18·0–82·0]	RIA (Diasorin)	58·6 (22·0)	5/223 (2·2%)	80·4 (21·5)	119:118 (237)	12·5 μg daily	Placebo	6 months	Lab confirmed influenza	223/237 (94·1%)
Lee 2018;^23^ USA	Children and young adults with sickle cell disease (30:32)	9·9 (3·9) [3·0–20·0]	LC-MS/MS, DEQAS	35·7 (16·5)	18/62 (29·0%)	92·4 (23·7)	31:31 (62)	2·5 mg bolus monthly	0·3 mg monthly	2 years	Self-reported respiratory events, including ARI	62/62 (100%)
Loeb 2018;^26^ Vietnam	Healthy children and adolescents (621:679)	8·5 (4·0) [3·0–17·0]	CLA (DiaSorin), DEQAS	65·5 (16·8)	5/1153 (0·4%)	91·8 (23·6)	650:650 (1300)	0·35 mg weekly	Placebo	8 months	RT-PCR confirmed influenza A or B	1153/1300 (88·7%)
Shimizu 2018^40^ Japan	Healthy adults (82:170)	53·1 (6·7) [45·0–74·0]	RIA (DiaSorin)	48·9 (13·5)	1/214 (0·5%)	114·6 (32·7)	126:126 (252)	10 μg daily (25[OH] D)‡	Placebo	4 months	URI: self-reported	215/252 (85·3%)
Ducharme 2022;^13^ Canada	Healthy adult healthcare workers (2:31)	40·0 (9·84) [25·0–58·0]	automated chemiluminescence analyzer, DiaSorin LIAISON XL platform	48·9 (21·9)	2/31 (6·5%)	97·7 (27·1)	18:15 (33)	2·5 mg bolus loading dose; then 0·25 mg weekly	Placebo	4 months	Lab confirmed COVID-19	33/34 (97·1%)
Huang 2022;^61^ Taiwan	Healthy preschool-age children (136:112)	3·9 (0·7) [range not reported]	Not reported	Not reported	0/21 (0·0%)	Not determined	135:113 (248)	50 μg daily	Placebo	6 months	Parent-reported influenza	248/248 (100%)
Reyes 2024;^63^ Chile	Healthy preschool children (168:135)	2·2 (0·5) [1·3–3·3]	LC-MS/MS	62·2 (15·5)	1/194 (0·5%)	0·44 mg group: 82·4 (24·5) 0·28 mg group: 104·6 (52·9)	99:103:101 (303)	0·14 mg/0·28 mg weekly	Placebo	6 months	ARI: self-reported	194/303 (64·0%)

**ARI co-primary outcome**												
Martineau 2015^33^ [ViDiCO]; UK	Adults with COPD (144:96)	64·7 (8·5) [40·0–85·0]	LC-MS/MS, DEQAS	46·1 (25·7)	50/240 (20·8%)	67·3 (27·5)	122:118 (240)	3 mg bolus 2-monthly	Placebo	1 year	URI: assessed from daily symptom diary	240/240 (100%)
Martineau 2015^34^ [ViDiAs]; UK	Adults with asthma (109:141)	47·9 (14·4) [16·0–78·0]	LC-MS/MS, DEQAS	49·6 (24·7)	36/250 (14·4%)	69·4 (21·0)	125:125 (250)	3 mg bolus 2-monthly	Placebo	1 year	URI: assessed from daily symptom diary	250/250 (100%)
Martineau 2015^32^ [ViDiFlu]; UK	Older adults and their carers (82:158)	67·1 (13·0) [21·4–94·0]	LC-MS/MS, DEQAS	42·9 (23·0)	60/240 (25·0%)	84·8 (24·1)	137:103 (240)	Older adults: 2·4 mg bolus 2-monthly + 10 μg daily Carers: 3 mg 2-monthly	Older adults: placebo + 10 μg daily Carers: placebo	1 year	URI & LRI, both assessed from daily symptom diary	240/240 (100%)
Gupta 2016;^18^ India	Children with pneumonia (226:98)	1·4 (1·1) [0·5–5·0]	RIA (Immunotech SAS/DiaSorin)	43·9 (33·4)	104/312 (33·3%)	64·1 (43·9)	162:162 (324)	2·5 mg bolus, single dose	Placebo	6 months	Physician confirmed recurrent pneumonia	314/324 (96·9%)
Bischoff-Ferrari 2020;^8^ Switzerland, France, Germany, Portugal, and Austria	Older adults (826:1331)	74·9 (4·4) [70·0–95·0]	LC-MS/MS, DEQAS	55·9 (21·0)	143/2140 (6·7%)	93·8 (28·2)	1076:1081	50 μg daily (2 × 2 × 2 factorial with omega-3 fatty acid supplementation and strength-training exercise)	Placebo	3 years	ARI: self-reported and verified by independent physician	2157/2157 (100%)

**ARI secondary outcome**												
Manaseki-Holland 2010;^29^ Afghanistan	Pre-school children with pneumonia (257:196)	1·1 (0·8) [0·1–3·3]	Not determined	Not determined	Not determined	Not determined	224:229 (453)	2·5 mg bolus once	Placebo	3 months	LRI: repeat episode of pneumonia-age-specific tachypnoea without wheeze	453/453 (100%)
Majak 2011^27^ Poland	Children with asthma (32:16)	10·9 (3·3) [6·0–17·0]	RIA (BioSource Europe), RIQAS	88·9 (38·2)	0/48 (0·0%)	37·6 (13·1)	24:24 (48)	12·5 μg daily	Placebo	6 months	ARI: self-report	48/48 (100%)
Trilok-Kumar 2011;^21^ India	Low birthweight infants (970:1109)	0·1 (0·0) [0·0–0·3]	..	Not determined	Not determined	55·0 (22·5)	1039:1040 (2079)	35 μg weekly	Placebo	6 months	ARI: medical record diagnosis of events causing hospitalisation	2064/2079 (99·3%)
Lehouck 2012;^24^ Belgium	Adults with COPD (145:37)	67·9 (8·3) [48·0–86·0]	RIA (Diasorin), DEQAS	49·8 (29·2)	31/182 (17·0%)	130·0 (44·7)	91:91 (182)	2·5 mg bolus monthly	Placebo	1 year	URI: self-report	175/182 (96·2%)
Camargo 2012;^9^ Mongolia	3^rd^/4^th^ grade schoolchildren (129:118)	10·0 (0·9) [7·0–12·7]	LC-MS/MS, DEQAS	18·9 (9·7)	192/245 (78·4%)	49·1 (15·1)	143:104 (247)	7·5 μg daily	Placebo	7 weeks	ARI: parent-reported ‘chest infections or colds’	244/247 (98·8%)
Bergman 2012;^7^ Sweden	Adults with increased susceptibility to ARI (38:102)	53·1 (13·1) [20·0–77·0]	CLA (DiaSorin), DEQAS	49·3 (23·2)	15/131 (11·5%)	94·9 (38·1)	70:70 (140)	100 μg daily	Placebo	1 year	URI: assessed with symptom score	124/140 (88·6%)
Rees 2013;^38^ USA	Adults with previous colorectal adenoma (438:321*)	61·2 (6·6) [47·1–77·9]	RIA (IDS), DEQAS	62·5 (21·3)	0/759 (0·0%)	186·9 (455·1)	399:360 (759)	25 μg daily	Placebo	13 months (average)	URI: assessed from daily symptom diary	759/759 (100%)
Tran 2014;^43^ Australia	Healthy older adults (343:301)	71·7 (6·9) [60·3–85·2]	CLA (DiaSorin), DEQAS	41·7 (13·5)	66/643 (10·3%)	71·0 (19·6)	430:214 (644)	0·75 mg bolus *vs* 1·5 mg bolus monthly	Placebo	1 year	URI: self-reported cold	594/644 (92·2%)
Grant 2014;^17^ New Zealand	Pregnant women and offspring 0:260 (pregnant women) 121:128 (offspring)	Offspring unborn at baseline	LC-MS/MS, DEQAS	54·8 (25·8)	30/200 (15·0%)	92·9 (41·6)	173:87 (pregnant women, 260) 164:85 (offspring, 249)	Pregnant women: 25 μg *vs* 50 μg daily. Offspring: 10 μg *vs* 20 μg daily	Placebo	9 months (3 months in pregnancy + 6 months in infancy)	ARI: doctor-diagnosed ARI precipitating primary care consult	236/260 (90·8%)
Denlinger 2016;^11^ USA	Adults with asthma (130:278)	39·2 (12·9) [18·0–85·0]	CLA (DiaSorin), VDSP	47·0 (16·9)	55/408 (13·5%)	104·3 (32·4)	201:207 (408)	2·5 mg bolus then 100 μg daily	Placebo	28 weeks	URI assessed with symptom score	408/408 (100%)
Tachimoto 2016;^42^ Japan	Children with asthma (50:39)	9·9 (2·3) [6·0–15·0]	RIA (DiaSorin), CAP	74·9 (24·6)	1/89 (1·1%)	85·7 (24·5)	54:35 (89)	20 μg daily, first 2 months	Placebo	6 months	URI: assessed with symptom score	89/89 (100%)
Hibbs 2018;^20^ USA	African American preterm infants (166:133†)	Offspring unborn at baseline	RIA	55·4 (22·2)	0/300 (0·0%)	95·0 (21·2)	153:147 (300)	10 μg daily, regardless of dietary intake	10 μg daily, only if dietary intake was <5 μg daily	1 year	ARI: self-reported URI/LRI	300/300 (100%)
Aloia 2019;^5^ USA	Healthy African American women aged over 60 years (0:260)	69·0 (5·3) [65·4–72·5]	LC-MS/MS, NIST	54·4 (16·7)	9/258 (3·5%)	117·0 (28·0)	130:130 (260)	50 μg daily	Placebo	3 months	ARI: self-reported cold/flu	260/260 (100%)
Hauger 2019;^19^ Denmark	Healthy children (61:69)	6·6 (1·5) [4·0–8·0]	LC-MS/MS, DEQAS	56·8 (12·5)	0/118 (0·0%)	20 μg group: 75·8 (11·5) 10 μg group: 61·8 (10·6)	43:44:43 (130)	20 μg/10 μg daily	Placebo	5 months	ARI: self-reported	118/130 (90·8%)
Camargo 2020;^10^ New Zealand	Older adults (2935:2121)	66·4 (8·3) [50·0–84·0]	LC-MS/MS, DEQAS	63·4 (23·6)	89/5056 (1·8%)	135·0 (39·9)	2558:2552 (5110)	5 mg bolus loading dose; then 2·5 mg bolus monthly	Placebo	3 years	ARI: self-reported cold/flu	5056/5110 (98·9%)
Ganmaa, 2020;^14^ Mongolia	Healthy school children (4485:4366)	9·4 (1·6) [6·0–13·0]	EIA (Biomerieux), DEQAS	29·7 (10·5)	2813/8851 (31·8%)	77·4 (22·7)	4418:4433 (8851)	0·35 mg weekly	Placebo	3 years	ARI: self-reported	8851/8851 (100%)
Mandlik 2020;^30^ India	Healthy children (158:127)	8·1 (1·2) [6·0–12·0]	EIA (DLD diagnostics)	58·9 (10·9)	0/237 (0·0%)	80 (23·3)	135:150 (285)	25 μg daily + 500 mg calcium	Placebo	6 months	URI: self-reported	244/285 (85·6%)
Pham 2020;^36^ Australia	Older adults (8678:7322)	69·3 (5·5) [60·0–86·0]	LC-MS/MS, VDSP	Not determined	Not determined	114·8 (30·3)§	8000:8000 (16000)	1·5 mg bolus monthly	Placebo	5 years	ARI: self-reported	16 000/16 000 (100%)
Rake 2020;^37^ England	Healthy older adults (408:379)	72·2 (4·9) [65·0–84·0]	CLA (Cobas 6000 Roche)	50·2 (27·1)	127/787 (16·1%)	109·2 (33·9)	395:392 (787)	2·5 mg bolus monthly	Placebo	2 years	URI/LRI: GP recorded	787/787 (100%)
Golan-Tripto unpublished;^64^ Israel	Prematurely born infants (21:29)	0 (0)	CLA (DiaSorin)	33·6 (29·7)	19/46 (41·3%)	20 μg group: 78·0 (75·0) 10 ug group: 81·0 (73·0)	25:25 (50)	20 μg daily	10 μg daily	1 year	ARI: GP recorded	25/50 (50·0%)
Camargo 2023;^62^ USA	Healthy older adults (7771:8033)	68·0 (7·0) [50·0–100·4]	LC-MS/MS	76·9 (25·0)	188/15804 (1·2%)	104·3 (29·6)	7905:7899 (15804)	50 μg daily (2 × 2 factorial with marine n-3 fatty acids)	Placebo	1 year	ARI: self-reported	15 013/15 804 (95·0%)

25(OH)D=25-hydroxyvitamin D. ARI=acute respiratory infection. CAP=College of American Pathologists. CLA=chemiluminescent assay. COPD=chronic obstructive pulmonary disease. D_3_=vitamin D_3_ (cholecalciferol). DEQAS=Vitamin D External Quality Assessment Scheme. EIA=enzyme immunoassay. EQA=external quality assessment. GP=general practitioner. ILI=influenza-like illness. IU=international units. LC-MS/MS=liquid chromatography tandem-mass spectrometry. LRI=lower respiratory infection. mo=month. RIA=radio-immunoassay. RIDT=rapid influenza diagnostic test. RIQAS=Randox International Quality Assessment Scheme. URI=upper respiratory infection. VDSP=Vitamin D Standardisation Program of the Office of Dietary Supplements, National Institutes of Health, USA. wk=week. yr=year.

* Sex missing for two participants randomised to intervention group and subsequently excluded from analysis due to lack of outcome data.

† Sex missing for one participant.

‡ Equivalent to 30 μg vitamin D_3·_ 1 μg vitamin D_3_=40 IU. 25(OH)D concentrations reported in ng/mL were converted to nmol/L by multiplying by 2·496.

§ From subset of participants randomised to intervention. For comparison, mean 25(OH)D at follow-up in subset of participants randomised to placebo was 77·5 nmol/L (SD 25·2 nmol/L).

**Table 2:** Proportion of participants in placebo controlled RCTs experiencing at least one ARI, overall and stratified by potential effect-modifiers

	No. of trials	Proportion with ≥1 ARI, intervention group (%)	Proportion with ≥1 ARI, control group (%)	Odds ratio (95% CI)	*I* ^2^	p for heterogeneity
Overall	40	15 202/31 092 (48·9%)	15 117/30 497 (49·6%)	0·94 (0·88–1·00)	26·4%	0·07

Baseline 25(OH)D, nmol/L*						
<25	22	1387/1893 (73·3%)	1408/1913 (73·6%)	0·98 (0·80–1·20)	3·6%	0·41
25–49·9	31	3783/5849 (64·7%)	3707/5769 (64·3%)	1·03 (0·94–1·13)	0·0%	0·55
50–74·9	32	2237/5749 (38·9%)	2142/5465 (39·2%)	0·90 (0·80–1·02)	8·7%	0·33
≥75	28	1530/6045 (25·3%)	1503/5899 (25·5%)	0·97 (0·87–1·07)	0·0%	0·83

Dosing frequency						
Daily	21	2572/10 920 (23·6%)	2569/10 632 (24·2%)	0·84 (0·73–0·97)	44·8%	0·014
Weekly	7	4483/6439 (69·6%)	4450/6350 (70·1%)	0·97 (0·88–1·06)	0·0%	0·44
Monthly or less frequently	12	8147/13 733 (59·3%)	8098/13 515 (59·9%)	0·98 (0·93–1·03)	0·0%	0·57

Daily dose equivalent, IU†						
<400	2	451/1074 (42·0%)	473/1059 (44·7%)	0·76 (0·41–1·41)	49·0%	0·16
400–1000	10	656/1236 (53·1%)	627/1069 (58·7%)	0·70 (0·55–0·89)	31·2%	0·16
1001–2000	19	11 494/24 790 (46·4%)	11 612/24 667 (47·1%)	0·97 (0·92–1·01)	1·6%	0·44
>2000	7	2291/3462 (66·2%)	2250/3444 (65·3%)	1·05 (0·84–1·31)	37·1%	0·15

Trial duration, months						
≤12	32	2847/12 615 (22·6%)	2766/12 063 (22·9%)	0·85 (0·76–0·95)	32·7%	0·040
>12	8	12 355/18 477 (66·9%)	12351/18 434 (67·0%)	0·99 (0·95–1·04)	0·0%	0·95

Age, years*						
<1	5	875/2901 (30·2%)	839/2796 (30·0%)	0·95 (0·82–1·10)	18·7%	0·30
1–15	16	4267/6028 (70·8%)	4271/5916 (72·2%)	0·74 (0·60–0·92)	33·2%	0·10
16–64	23	3428/7323 (46·8%)	3413/7175 (47·6%)	0·95 (0·86–1·05)	12·9%	0·29
≥65	18	6631/14 907 (44·5%)	6611/14 676 (45·0%)	0·97 (0·92–1·02)	0·0%	0·78

Airway disease						
Asthma only	4	203/404 (50·2%)	202/391 (51·7%)	0·73 (0·36–1·49)	71·7%	0·014
COPD only	2	106/208 (51·0%)	104/207 (50·2%)	1·01 (0·68–1·51)	0·0%	0·71
Unrestricted	34	14 893/30 480 (48·9%)	14 811/29 899 (49·5%)	0·94 (0·89–1·00)	26·4%	0·14

ARI=acute respiratory infection. COPD=chronic obstructive pulmonary disease. RCT=randomised controlled trial.

* The number of trials in each category for this variable adds up to more than 40, since this is a participant-level variable (ie, some trials contributed data from participants who fell into more than one category).

† Data from two trials that included higher-dose, lower-dose, and placebo groups^43,63^ are excluded from this sub-group analysis, since the higher-dose and lower-dose groups spanned the 1000 IU/day cut-off, rendering them unclassifiable.

**Table 3:** Secondary outcomes of placebo-controlled studies

	No. of trials	Proportion with ≥1 event, intervention group (%)	Proportion with ≥1 event, control group (%)	Odds ratio (95% CI)	*I* ^2^	p for heterogeneity
**Efficacy outcomes**						
Upper respiratory infection*	32	9396/22 244 (42·2%)	9341/21 734 (43·0%)	0·95 (0·91–1·01)	4·6%	0·39
Lower respiratory infection*	16	4040/20 915 (19·3%)	4049/20 739 (19·5%)	0·99 (0·93–1·04)	0·0%	0·58
Emergency department attendance and/or hospital admission due to ARI	20	139/10 981 (1·3%)	149/10 865 (1·4%)	0·90 (0·71–1·14)	0·0%	1·00
Death due to ARI or respiratory failure	35	14/14 706 (0·1%)	11/14 154 (0·1%)	1·03 (0·61–1·75)	0·0%	1·00
Use of antibiotics to treat an ARI*	15	2056/8656 (23·8%)	2109/8519 (24·8%)	0·93 (0·86–1·01)	2·0%	0·43
Absence from work or school due to ARI	11	378/1545 (24·5%)	364/1059 (34·4%)	0·91 (0·70–1·18)	28·1%	0·18

**Safety outcomes**						
Serious adverse event of any cause*	38	1579/22 860 (6·9%)	1621/22 321 (7·3%)	0·96 (0·90–1·04)	0·0%	1·00
Death due to any cause	37	438/22 853 (1·9%)	397/22 288 (1·8%)	1·09 (0·95–1·25)	0·0%	1·00
Hypercalcaemia	23	143/18 275 (0·8%)	124/17 899 (0·7%)	1·13 (0·89–1·44)	0·0%	1·00
Renal stones	23	415/20 539 (2·0%)	401/20 133 (2·0%)	1·03 (0·90–1·18)	0·0%	1·00

ARI=acute respiratory infection.

* This analysis includes a subset of participants in the trial by Pham and colleagues, who completed symptom diaries.

## Data Availability

The study dataset is available upon request to the corresponding author (d.a.jolliffe@qmul.ac.uk).

## Research in context

### Evidence before this study

Our previous meta-analysis of 43 RCTs of vitamin D supplementation for prevention of acute respiratory infections (ARI) conducted in 2021 revealed a statistically significant protective effect of the intervention (odds ratio [OR] 0·92 [95% CI 0·86–0·99). We searched MEDLINE, EMBASE, the Cochrane Central Register of Controlled Trials, Web of Science, Science Direct, and the ClinicalTrials.gov registry from May 1, 2020 (date of our previous search) to April 30, 2024 for randomised controlled trials (RCTs) and meta-analyses of randomised controlled trials evaluating effectiveness of vitamin D supplementation for the prevention of acute respiratory infections. A further six eligible RCTs, contributing data from 19 337 participants have now been completed, including one large trial (n=15 804).

### Added value of this study

Our meta-analysis of aggregate data from 64 086 participants in 46 RCTs, stratified by baseline 25(OH)D concentration and age, provides an updated estimate of the effects of vitamin D on ARI overall (OR 0·94 [95% CI 0·88–1·00), and in subgroups defined by baseline vitamin D status, age, dosing frequency, amount, and duration.

### Implications of all the available evidence

Updated meta-analysis including the latest available RCT data shows no statistically significant protective effect of vitamin D supplementation against ARI, either overall or in subgroup analyses.
