# Supplementary material for: Vitamin D supplementation to prevent acute respiratory infections: systematic review and meta-analysis of stratified aggregate data
Source: Lancet Diabetes Endocrinol. Author manuscript; Available in PMC 2025 May 7. (PMC12056739; doi:10.1016/S2213-8587(24)00348-6)
Supplement: Supplementary [file NIHMS2078206-supplement-Supplementary.pdf]

THE LANCET
Diabetes & Endocrinology

Supplementary appendix

This appendix formed part of the original submission and has been peer reviewed. We post it as supplied by the authors.

Supplement to: Joliffe DA, Camargo CA Jr, Sluuyter JD, et al. Vitamin D supplementation to prevent acute respiratory infections: systematic review and meta-analysis of stratified aggregate data. *Lancet Diabetes Endocrinol* 2025; published online Feb 21. https://doi.org/10.1016/S2213-8587(24)00348-6.

Vitamin D supplementation to prevent acute respiratory infections: systematic review and meta-analysis of stratified aggregate data

Appendix

Table of Contents

1 Search strategies....4
2 Data Collection Processes....6
3 Sources of support for participating trials....7
4 Registration and Ethical Approvals....9
5 Measure of effect and exploration of variation in effects....10
6 Appendix Table 1: Reasons for exclusion of potentially relevant studies....11
7 Appendix Table 2: Risk of Bias Assessment....12
8 Appendix Table 3: Multivariable meta-regression model for proportion of participants experiencing at least one acute respiratory tract infection, by trial-level subgroups....14
9 Appendix Table 4: Summary of Findings Table....15
10 Appendix Table 5: Results of exploratory sensitivity analyses excluding placebo-controlled trials at unclear risk of bias and studies investigating ARI incidence as a secondary outcome....16
11 Appendix Table 6: Sensitivity analysis of sub-groups with data from ≤ 5 trials, using the Hartung-Knapp-Sidik-Jonkman model....17
12 Appendix Figure 1: Cates plot illustrating reduction in risk of one or more acute respiratory infections with vitamin D supplementation vs. placebo, overall (primary comparison)....18
13 Appendix Figure 2: Forest plot of RCTs comparing effects of higher- vs. lower-dose vitamin D, reporting proportion of participants experiencing at least one acute respiratory infection....19
14 Appendix Figure 3: Forest plot of RCTs comparing effects of vitamin D vs. placebo, reporting proportion of participants experiencing at least one acute respiratory infection, by baseline 25-hydroxyvitamin D level. A) <25.0 nmol/L; B) 25.0 to 49.9 nmol/L; C) 50.0 to 74.9 nmol/L, and D) ≥75.0 nmol/L. 20
15 Appendix Figure 4: Forest plot of RCTs comparing effects of vitamin D vs. placebo, reporting proportion of participants experiencing at least one acute respiratory infection, by frequency of supplementation (daily vs. weekly vs. monthly to 3-monthly)....21
16 Appendix Figure 5: Forest plot of RCTs comparing effects of vitamin D vs. placebo, reporting proportion of participants experiencing at least one acute respiratory infection, by daily dose equivalents (<400 IU/day vs. 400-1000 IU/day vs. 1001-2000 IU/day vs. >2000 IU/day)....22
17 Appendix Figure 6: Forest plot of RCTs comparing effects of vitamin D vs. placebo, reporting proportion of participants experiencing at least one acute respiratory infection, by trial duration (≤12 months vs. >12 months)....23
18 Appendix Figure 7: Forest plot of RCTs comparing effects of vitamin D vs. placebo, reporting proportion of participants experiencing at least one acute respiratory infection, by the following age strata: A) <1 year; B) 1-15.99 years; C) 16-64.99 years, and D) ≥65 years....24
19 Appendix Figure 8: Forest plot of RCTs comparing effects of vitamin D vs. placebo, reporting proportion of participants experiencing at least one acute respiratory infection, by presence or absence of airway disease comorbidity....25
20 Appendix Figure 9: Funnel plot of placebo-controlled RCTs reporting proportion of participants experiencing 1 or more acute respiratory infection....26
21 References....28

1 Search strategies

A. Medline

Cochrane Highly Sensitive Search Strategy for identifying randomised controlled trials

#1. randomized controlled trial [pt] OR controlled clinical trial [pt] OR randomized [tiab] OR placebo [tiab] OR drug therapy [sh] OR randomly [tiab] OR trial [tiab] OR groups [tiab]

#2. animals [mh] NOT humans [mh]

#3. #1 NOT #2

Terms specific to vitamin D

#4. Vitamin D OR vitamin D2 OR vitamin D3 OR cholecalciferol OR ergocalciferol OR alphacalcidol OR alfacalcidol OR calcitriol OR paricalcitol OR doxerocalciferol

Terms specific to acute respiratory infection

#5. Acute Respiratory Infection OR Upper Respiratory Infection OR Lower Respiratory Infection OR Respiratory Tract Infection OR Common Cold OR Sinusitis OR Pharyngitis OR Laryngitis OR Laryngotracheobronchitis OR Tonsillitis OR peritonsillar abscess OR Croup OR Epiglottitis OR supraglottitis OR Otitis Media OR Pneumonia OR Bronchopneumonia OR Bronchitis OR Bronchiolitis OR Pleurisy OR Pleuritis OR Wheez* OR Respiratory OR Asthma

Combination of terms to identify randomised controlled trials of vitamin D for the prevention of acute respiratory infection

#3 AND #4 AND #5

B. EMBASE

Terms for identifying randomised controlled trials

#1. ‘randomized controlled trial’/exp OR ‘single blind procedure’/exp OR ‘double blind procedure’/exp OR ’crossover procedure’/exp

#2. random*:ab,ti OR placebo*:ab,ti OR crossover*:ab,ti OR ’cross over’:ab,ti OR allocat*:ab,ti OR ((singl* OR doubl*) NEXT/1 blind*):ab,ti OR trial:ti

#3. #1 OR #2

Terms specific to vitamin D

#4. *vitamin AND d OR vitamin AND d2 OR vitamin AND d3 OR cholecalciferol OR ergocalciferol OR alphacalcidol OR alfacalcidol OR calcitriol OR paricalcitol OR doxerocalciferol*

Terms specific to acute respiratory infection

#5. acute AND respiratory AND infection OR upper AND respiratory AND infection OR lower AND respiratory AND infection OR respiratory AND tract AND infection OR common AND cold OR sinusitis OR pharyngitis OR laryngitis OR laryngotracheobronchitis OR tonsillitis OR peritonsillar AND abscess OR croup OR epiglottitis OR supraglottitis OR otitis AND media OR pneumonia OR bronchopneumonia OR bronchitis OR bronchiolitis OR pleurisy OR pleuritis OR wheez* OR respiratory OR asthma

Combination of terms to identify randomised controlled trials of vitamin D for the prevention of acute respiratory infection

#3 AND #4 AND #5

C. Cochrane Central

Terms specific to vitamin D

#1. Vitamin D OR vitamin D2 OR vitamin D3 OR cholecalciferol OR ergocalciferol OR alphacalcidol OR alfacalcidol OR calcitriol OR paricalcitol OR doxerocalciferol

Terms specific to acute respiratory infection

#2. Acute Respiratory Infection OR Upper Respiratory Infection OR Lower Respiratory Infection OR Respiratory Tract Infection OR Common Cold OR Sinusitis OR Pharyngitis OR Laryngitis OR Laryngotracheobronchitis OR Tonsillitis OR peritonsillar abscess OR Croup OR Epiglottitis OR supraglottitis OR Otitis Media OR Pneumonia OR Bronchopneumonia OR Bronchitis OR Bronchiolitis OR Pleurisy OR Pleuritis OR Wheez* OR Respiratory OR Asthma

Combination of terms to identify randomised controlled trials of vitamin D for the prevention of acute respiratory infection

#1 AND #2

D. Web of Science

TS =(Vitamin D OR vitamin D2 OR vitamin D3 OR cholecalciferol OR ergocalciferol OR alphacalcidol OR alfacalcidol OR calcitriol OR paricalcitol OR doxerocalciferol) AND TS =(Acute Respiratory Infection OR Upper Respiratory Infection OR Lower Respiratory Infection OR Respiratory Tract Infection OR Common Cold OR Sinusitis OR Pharyngitis OR Laryngitis OR Laryngotracheobronchitis OR Tonsillitis OR peritonsillar abscess OR Croup OR Epiglottitis OR supraglottitis OR Otitis Media OR Pneumonia OR Bronchopneumonia OR Bronchitis OR Bronchiolitis OR Pleurisy OR Pleuritis OR Wheez* OR Respiratory OR Asthma) AND TS =(placebo* or random* or clinical trial* or double blind* or single blind* or rct)

E. Science Direct

(Vitamin D OR cholecalciferol OR ergocalciferol OR calcitriol) AND (respiratory infection OR pneumonia OR bronchitis OR common cold OR influenza)

F. ClinicalTrials.gov

Vitamin D AND respiratory AND infection

2 Data Collection Processes

Summary data from trials which contributed to our previous meta-analysis of individual participant data,[1] were extracted from our central database with permission from the Principal Investigators. Summary data relating to the primary outcome (overall and by sub-group) and secondary outcomes (overall only) from newly identified trials were requested from Principal Investigators. On receipt, they were assessed for consistency with associated publications. Study authors were contacted to provide missing data and to resolve any queries arising from these consistency checks. Once queries had been resolved, clean summary data were uploaded to the study database, which was held in STATA IC v14.2 (StataCorp, College Station, TX).

Data were extracted for the following variables: study setting, eligibility criteria, age, sex and baseline vitamin D status of participants, details of intervention and control regimens, trial duration, case definitions for ARI, and numbers of participants contributing data to statistical analyses. Follow-up summary data were requested for the proportions of participants experiencing one or more ARI during the trial, both overall and stratified by potential effect modifiers, where this was available. We also requested summary data on the proportions of participants who experienced one or more of the following events during the trial: upper respiratory infection (URI); lower respiratory infection (LRI);

Emergency Department attendance and/or hospital admission for ARI; death due to ARI or respiratory failure; use of antibiotics to treat an ARI; absence from work or school due to ARI; a serious adverse event; death due to any cause; and potential adverse reactions to vitamin D (hypercalcaemia and renal stones).

3 Sources of support for participating trials

The trial by Aglipay and colleagues was supported by the competitive grants from the Canadian Institutes of Health Research Institutes of Human Development, Child and Youth Health and Nutrition, Metabolism and Diabetes (grant number MOP-114945) and the Thrasher Research Fund (award number 9113).

The trial by Aloia and colleagues was supported by the National Institute of Aging (grant number R01-AG032440-01A2).

The trial by Arihiro and colleagues was supported by the Ministry of Education, Culture, Sports, Science, and Technology in the Japan-Supported Program for the Strategic Research Foundation at Private Universities and funding from the Department of Gastroenterology and Hepatology, Jikei University of Medicine, Tokyo, Japan.

The trial by Bergman and colleagues was supported by grants from the Swedish Research Council, the Strategic Research Foundation (SSF), the Swedish Heart and Lung foundation, Karolinska Institutet, Stockholm County Council and the Swedish Cancer Society as well as by the Magnus Bergwall and Åke Wiberg foundations.

The trial by Bischoff-Ferrari and colleagues was supported by grants from the Seventh Framework Program of the European Commission (grant agreement 278588), the University of Zurich (Chair for Geriatric Medicine and Aging Research), DSM Nutritional Products, Roche, NESTEC, Pfizer, and Streuli.

The trials by Camargo and colleagues were supported by a grant for the Blue Sky Study from an anonymous foundation and the Massachusetts General Hospital; and the Health Research Council of New Zealand (grant number 10/400) and the Accident Compensation Corporation of New Zealand. The trial by Camargo, Manson, and colleagues was supported by National Institutes of Health (NIH) (grant numbers U01 CA138962, R01 CA138962, R01 AT011729, R01 AI093723, K24 HL136852). Pharmavite, Pronova BioPharma/BASF, and Quest Diagnostics provided study medications and diagnostic services.

The trial by Ganmaa and colleagues was supported by the National Institutes of Health (Grant Number 1R01HL122624-01).

The trial by Ginde and colleagues was supported by NIH/NIA grant K23AG040708, NIH/NCATS Colorado CTSA Grant UL1TR001082, and the American Geriatrics Society Jahnigen Career Development Scholars Award.

The trial by Golan-Tripto and colleagues was supported by the Soroka JNF UK Clinical Research Scholar Program.

The trial by Goodall and colleagues was supported in part by the Canadian Institutes of Health Research [OPP 86940] and with in-kind support from Copan Italia, Bresica Italy.

The trial by Grant and colleagues was supported by the Health Research Council of New Zealand, Grant Number 09/215R.

The trial by Gupta and colleagues was supported by the Indian Council of Medical Research, New Delhi.

The trial by Hauger and colleagues was supported by Lundbeckfonden (grant number R180-2014-3481), by Brødrene Hartmann's Fund (A26842), and by the European Commission (FP7/2007–2013) under Grant Agreement 613977 for the ODIN Integrated Project (Food-based solutions for optimal vitamin D nutrition and health through the life cycle).

The trial by Hibbs and colleagues was supported by the National Heart, Lung, and Blood Institute and Office of Dietary Supplements (grant number R01HL109293).

The trial by Lee and colleagues was supported by the US Food and Drug Administration Orphan Product Development (grant number R01FD003894).

The trial by Loeb and colleagues was supported by the Institute for Infectious Diseases Research at McMaster University.

The trials by Manaseki-Holland and colleagues were supported by New Zealand Aid (ref: GRA/470/2) and The Wellcome Trust (ref: 082476).

The trial by Mandlik and colleagues was supported by a core grant from the Hirabai Cowasji Jehangir Medical Research Institute.

The trials by Martineau and colleagues were supported by the National Institute for Health Research under its Programme Grants for Applied Research Programme (Reference Number RP-PG-0407-10398).

The trial by Murdoch and colleagues was supported by the Health Research Council of New Zealand, grant number 09/302.

The trial by Pham and colleagues was supported by project grants from the National Health and Medical Research Council (grant numbers GNT1046681 and GNT1120682).

The trial by Reyes and colleagues was supported by Fondo Nacional de Investigacion y Desarrollo en Salud (ref. SA13I20173).

The trial by Rosendahl and colleagues was supported by the Foundation for Pediatric Research, the Finnish Medical Foundation, Governmental Subsidy for Clinical Research, the Päivikki and Sakari Sohlberg Foundation, the Academy of Finland, the Sigrid Jusélius Foundation, the Folkhälsan Research Foundation, the Novo Nordisk Foundation, the Orion Research Foundation, and Barncancerfonden.

The trial by Rake and colleagues was supported by the National Institute for Health Research Health Technology Assessment programme (ref. HTA 08/116/48).

The trial by Rees and colleagues was supported by the National Cancer Institute at the National Institutes of Health (grant numbers CA098286 and CA098286-S).

The trial by Shimizu and colleagues was supported by the FANCL Corporation.

The trial by Simpson and colleagues was supported by the Royal Hobart Hospital Research Foundation.

The trial by Tachimoto and colleagues was supported by the Ministry of Education, Culture, Sports, Science, and Technology in the Japan-Supported Program for the Strategic Research Foundation at Private Universities, JSPH KAKENHI Grant Number 23591553, and funding from Jikei University of Medicine, Tokyo, Japan.

The trial by Tran and colleagues was supported by the National Health and Medical Research Council of Australia, grant 613655.

The trial by Trilok Kumar and colleagues was supported by the Department of Biotechnology, Government of India (ref BT/PR-PR7489/PID/20/285/2006), Nutrition Third World and Sight and Life

4 Registration and Ethical Approvals

Research Ethics Committee approval to conduct this meta-analysis was not required in the UK; local ethical permission to contribute data from primary trials was required and obtained for studies by Camargo *et al,*[2] (The Ethics Review Committee of the Mongolian Ministry of Health), Murdoch *et al,*[3] (Southern Health and Disability Ethics Committee, ref. URB/09/10/050/AM02), Rees *et al,*[4] (Committee for the Protection of Human Subjects, Dartmouth College, USA; Protocol # 24381), Tachimoto *et al,*[5] (Ethics committee of the Jikei University School of Medicine, ref 26-333: 7839), Tran *et al,*[6] (QIMR Berghofer

Medical Research Institute Human Research Ethics Committee, P1570) and Urashima *et al*,[7,8] (Ethics committee of the Jikei University School of Medicine, ref 26-333: 7839).

5 Measure of effect and exploration of variation in effects

Odds ratios were pre-specified as the effects measure in all analyses in our study protocol, in order to avoid potential pitfalls when using risk ratios in meta-analyses.[9] This approach is entirely in accordance with the Cochrane Handbook's guidelines[10]. It also allows readers to make a direct comparison of results from the current analysis with those of our previous meta-analyses, which also used this methodology.[1,11-13] To investigate factors associated with heterogeneity of effect between statistically significant subgroups of trials, we performed multivariable meta-regression analysis on trial-level characteristics, namely, dose frequency, dose size, trial duration and age at enrolment, to produce an adjusted odds ratio, a 95% CI and a P value for interaction for each factor. This was conducted using the STATA package, 'metareg' - a variance-weighted least squares (VWLS) method with a tau-squared (τ^2) variance component. The model response variable was the natural logarithm of the odds ratio for the outcome of each trial, and the random error distribution was assumed to be independently and normally distributed, with variance equal to the squared within-study standard error of the log OR. Independent variables were dichotomised to create a more parsimonious model (baseline serum 25(OH)D of <25 vs. ≥25 nmol/L; administration of daily vs. non-daily doses; daily equivalent of ≤1000 IU vs. >1000 IU; trial duration of ≤12 vs. >12 months, and participant age of <16.00 vs. ≥16.00 years at enrolment). The meta-regression analysis excluded data from two placebo-controlled trials that included higher-dose, lower-dose and placebo arms,[6,14] (since the higher-dose and lower-dose arms in these studies spanned the 1,000 IU/day cut-off), and four placebo-controlled trials that enrolled participants aged below and above the age cut-off of 16 years.[7,15-17] These factors rendered these trials unclassifiable for the purposes of the meta-regression analysis.

6 Appendix Table 1: Reasons for exclusion of potentially relevant studies

**First author, year or clinicaltrials.gov registration number**	**Reason for exclusion**
Somnath, 2017[18]	Ineligible: open-label trial
Jung, 2018[19]	Ineligible: ARI outcome not pre-specified
Ramos-Martínez, 2018[20]	Ineligible: intervention was administration of 1,25-dihydroxyvitamin D
Zhou, 2018[21]	Ineligible: open-label trial
Hueniken, 2019[22]	Ineligible: same trial as Aglipay et al[23]
Singh, 2019[24]	Ineligible: open-label trial
Jolliffe, 2022[25]	Ineligible: open-label trial
van Helmond[26]	Ineligible: open-label trial
NCT05037253	Ineligible: open-label trial
NCT04810949	Ineligible: open-label trial
Villasis-Keever[27]	Eligible: unresponsive to invitation to contribute data
NCT04386850	Potentially eligible: status of completion could not be determined
NCT03956732	Ineligible: second intervention administered to vitamin D arm

7 Appendix Table 2: Risk of Bias Assessment

	Sequence generation	Allocation concealment	Blinding of participants and personnel	Blinding of outcome assessment	Incomplete outcome data	Selective reporting	Other bias
Li-Ng 2009[28]	✓	✓	✓	✓	✓	✓	✓
Urashima 2010[8]	✓	✓	✓	✓	✓	✓	✓
Manaseki-Holland 2010[29]	✓	✓	✓	✓	✓	✓	✓
Laaksi 2010[30]	✓	✓	✓	✓	?	✓	✓
Majak 2011[15]	✓	✓	✓	✓	✓	✓	✓
Trilok-Kumar 2011[31]	✓	✓	✓	✓	✓	✓	✓
Lehouck 2012[32]	✓	✓	✓	✓	✓	✓	✓
Manaseki-Holland 2012[33]	✓	✓	✓	✓	✓	✓	✓
Camargo 2012[2]	✓	✓	✓	✓	✓	✓	✓
Murdoch 2012[3]	✓	✓	✓	✓	✓	✓	✓
Bergman 2012[34]	✓	✓	✓	✓	✓	✓	✓
Marchisio 2013[35]	✓	✓	✓	✓	✓	✓	✓
Rees 2013[4]	✓	✓	✓	✓	✓	✓	✓
Tran 2014[6]	✓	✓	✓	✓	✓	✓	✓
Goodall 2014[36]	✓	✓	✓	✓	✓	✓	✓
Urashima 2014[7]	✓	✓	✓	✓	✓	✓	✓
Grant 2014[37]	✓	✓	✓	✓	✓	✓	✓
Martineau 2015a[38] [ViDiCO]	✓	✓	✓	✓	✓	✓	✓
Martineau 2015b[39] [ViDiAs]	✓	✓	✓	✓	✓	✓	✓
Martineau 2015c[40] [ViDiFlu]	✓	✓	✓	✓	✓	✓	✓
Simpson 2015[41]	✓	✓	✓	✓	✓	✓	✓
Dubnov-Raz 2015[16]	✓	✓	✓	✓	?	✓	✓
Denlinger 2016[42]	✓	✓	✓	✓	✓	✓	✓
Tachimoto 2016[5]	✓	✓	✓	✓	✓	✓	✓
Ginde 2016[43]	✓	✓	✓	✓	✓	✓	✓
Gupta 2016[44]	✓	✓	✓	✓	✓	✓	✓
Aglipay 2017[23]	✓	✓	✓	✓	✓	✓	✓
Arihiro 2018[45]	✓	✓	✓	✓	✓	✓	✓
Hibbs 2018[46]	✓	✓	✓	✓	✓	✓	✓
Lee 2018[47]	✓	✓	✓	✓	✓	✓	✓
Loeb 2018[17]	✓	✓	✓	✓	✓	✓	✓
Rosendahl 2018[48]	✓	✓	✓	✓	✓	✓	✓
Shimizu 2018[49]	✓	✓	✓	✓	✓	✓	✓
Aloia 2019[50]	✓	✓	✓	✓	✓	✓	✓
Hauger 2019[51]	✓	✓	✓	✓	✓	✓	✓
Bischoff-Ferrari[52]	✓	✓	✓	✓	✓	✓	✓
Camargo 2020[53]	✓	✓	✓	✓	✓	✓	✓
Ganmaa 2020[54]	✓	✓	✓	✓	✓	✓	✓
Mandlik 2020[55]	✓	✓	✓	✓	✓	✓	✓
Pham 2020[56]	✓	✓	✓	✓	✓	✓	✓
Rake 2020[57]	✓	✓	✓	✓	✓	✓	✓
Ducharme 2022[58]	✓	✓	✓	✓	✓	**N/A**	✓
Huang 2022[59]	✓	✓	✓	?	?	?	✓
Camargo 2023[60]	✓	✓	✓	✓	✓	✓	✓
Reyes 2024[61]	✓	✓	✓	✓	?	**N/A**	✓
Golan-Tripto (unpublished)[62]	✓	✓	✓	✓	?	**N/A**	✓

✓ = low risk of bias; ? = unclear risk of bias, N/A = not applicable (unpublished)

8 Appendix Table 3: Multivariable meta-regression model for proportion of participants experiencing at least one acute respiratory tract infection, by trial-level subgroups.

Variables	No of trials[a]	Proportion with ≥1 ARI, intervention group (%)	Proportion with ≥1 ARI, control group (%)	Odds ratio (95% CI)[b]	I^2 %	P value for heterogeneity	Adjusted odds ratio (95% CI)[c]	P value for interaction[c]
**Dosing frequency**								
Daily	21	2572/10920 (23.6)	2569/10632 (24.2)	0.84 (0.73 to 0.97)	44.8	0.014	1.01 (0.87 to 1.17)	0.91
Not daily	18	12445/19775 (62.9)	12452/19668 (63.3)	0.98 (0.93 to 1.02)	0.0	0.56	Referent	
**Daily dose equivalent, IU**								
≤1000	12	1107/2310 (47.9)	1100/2128 (51.7)	0.74 (0.60 to 0.90)	35.3	0.11	0.89 (0.73 to 1.08)	0.23
>1000	26	13785/28252 (48.8)	13862/28111 (49.3)	0.98 (0.93 to 1.04)	14.7	0.25	Referent	
**Trial duration, months**								
≤12	31	2847/12615 (22.6)	2766/12063 (23.0)	0.85 (0.76 to 0.95)	32.7	0.040	Referent	0.20
>12	8	12355/18477 (66.9)	12351/18434 (67.0)	0.99 (0.95 to 1.04)	0.0	0.95	1.10 (0.95 to 1.28)	
**Age, years**								
≥16	21	9845/21674 (45.4)	9914/21545 (46.0)	0.96 (0.90 to 1.03)	17.6	0.23	0.99 (0.86 to 1.14)	0.86
<16	14	5076/8258 (61.5)	5026/8045 (62.5)	0.94 (0.88 to 1.00)	38.5	0.07	Referent	

[a] Data from two trials that included higher-dose, lower-dose and placebo arms[6,14] are excluded, since the higher-dose and lower-dose arms in these studies spanned the 1,000 IU/day cut-off, and data from four placebo-controlled trials [7,15-17]were excluded, since some participants spanned the 16 years of age cut-off, rendering all these trials unclassifiable for the purposes of this analysis.
[b] Within-sub-group odds ratios from random effects model adjusting for study weights.
[c] adjusted odds ratios and P values for interaction from multivariable meta-regression model including dichotomised variables for dose frequency, size and trial duration.

9 Appendix Table 4: Summary of Findings Table

Vitamin D_3 compared to placebo for prevention of acute respiratory infection (ARI)

Population: children and adults of any age, sex or ethnic origin, with or without co-morbidity
Setting: Eighteen countries on four continents (Asia, Australasia, Europe, North America)
Intervention: oral vitamin D_3 (cholecalciferol) supplementation
Comparison: oral placebo

Outcomes	Anticipated absolute effects* (95% CI)		Relative effect (95% CI)	№ of participants (studies)	Quality of the evidence (GRADE)
	**Risk with placebo**	**Risk with Vitamin D**			
Proportion with at least one ARI, all participants	496 per 1,000	480 per 1,000 (464 to 496)	OR 0.94 (0.88 to 1.00)	61589 (40 RCTs)	⊕⊕⊕ MODERATE
Proportion with at least one ARI, participants in placebo-controlled trials with duration ≤12 months investigating daily dosing with 400-1,000 IU vitamin D_3/day	572 per 1,000	436 per 1,000 (375 to 500)	OR 0.58 (0.45 to 0.75)	1232 (8 RCTs)	⊕⊕⊕ MODERATE
Proportion with at least one hospital admission or emergency department attendance due to ARI	14 per 1,000	12 per 1,000 (10 to 16)	OR 0.90 (0.71 to 1.14)	21846 (20 RCTs)	⊕⊕⊕ MODERATE
Proportion with serious adverse event, any cause	73 per 1,000	70 per 1,000 (66 to 75)	OR 0.96 (0.90 to 1.04)	45181 (38 RCTs)	⊕⊕⊕ MODERATE
Proportion of deaths due to ARI or respiratory failure	1 per 1,000	1 per 1,000 (0 to 1)	OR 1.03 (0.61 to 1.75)	28860 (35 RCTs)	⊕⊕⊕ MODERATE

*__The risk in the intervention group__ (and its 95% confidence interval) is based on the assumed risk in the comparison group and the **relative effect** of the intervention (and its 95% CI).

CI: Confidence interval; **OR:** Odds ratio

GRADE Working Group grades of evidence
High quality: We are very confident that the true effect lies close to that of the estimate of the effect
Moderate quality: We are moderately confident in the effect estimate: The true effect is likely to be close to the estimate of the effect, but there is a possibility that it is substantially different
Low quality: Our confidence in the effect estimate is limited: The true effect may be substantially different from the estimate of the effect
Very low quality: We have very little confidence in the effect estimate: The true effect is likely to be substantially different from the estimate of effect

10 Appendix Table 5: Results of exploratory sensitivity analyses excluding placebo-controlled trials at unclear risk of bias and studies investigating ARI incidence as a secondary outcome.

Reason for exclusion (number of excluded studies)	No. of studies included	Proportion with ≥1 ARI, intervention group (%)	Proportion with ≥1 ARI, control group (%)	Odds ratio (95% CI)	I^2 %	P value for heterogeneity
Studies at unclear risk of bias (n=4)	36	15027/30730 (48.9)	14989/30228 (49.6)	0.95 (0.90 to 1.01)	22.3	0.12
Studies with ARI incidence as secondary outcome (n=19)	21	1804/5080 (35.5)	1767/4895 (36.1)	0.90 (0.79 to 1.02)	18.2	0.22
Studies designed to detect an effect of vitamin D on recurrent ARI (n=3)	37	15040/30654	14917/30052	0.96 (0.91 to 1.02)	15.9	0.20

11 Appendix Table 6: Sensitivity analysis of sub-groups with data from ≤ 5 trials, using the Hartung-Knapp-Sidik-Jonkman model.

Potential effect-modifier	No of trials	Proportion with ≥1 ARI, intervention group (%)	Proportion with ≥1 ARI, control group (%)	Odds ratio (95% CI)	I^2 %	P for heterogeneity
**Daily dose equivalent, IU**						
<400 [a]	2	451/1074 (42.0)	473/1059 (44.7)	0.76 (0.41 to 1.41)	49.0	0.16
<400 [b]	2	451/1074 (42.0)	473/1059 (44.7)	0.76 (0.01 to 40.91)	49.0	0.16
**Age, yrs**						
<1.00 [a]	5	875/2901 (30.2)	839/2796 (30.0)	0.95 (0.82 to 1.10)	18.7	0.30
<1.00 [b]	5	875/2901 (30.2)	839/2796 (30.0)	0.95 (0.77 to 1.18)	18.7	0.30
**Airway disease**						
Asthma only [a]	4	203/404 (50.2)	202/391 (51.7)	0.73 (0.36 to 1.49)	71.7	0.014
Asthma only [b]	4	203/404 (50.2)	202/391 (51.7)	0.73 (0.22 to 2.39)	71.7	0.014
COPD only [a]	2	106/208 (51.0)	104/207 (50.2)	1.01 (0.68 to 1.51)	0.0	0.71
COPD only [b]	2	106/208 (51.0)	104/207 (50.2)	1.01 (0.08 to 13.70)	0.0	0.71

[a] Estimates from DerSimonian-Laird models, presented in Table 2.
[b] Estimates from Hartung-Knapp-Sidik-Jonkman model.

12 Appendix Figure 1: Cates plot illustrating reduction in risk of one or more acute respiratory infections with vitamin D supplementation vs. placebo, overall (primary comparison).

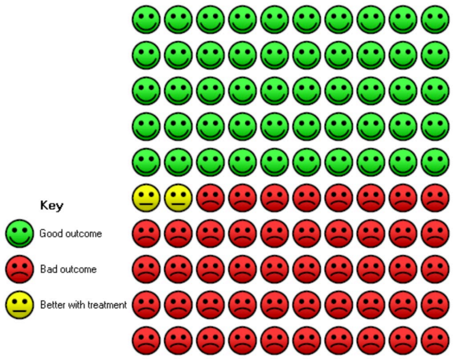

13 Appendix Figure 2: Forest plot of RCTs comparing effects of higher- vs. lower-dose vitamin D, reporting proportion of participants experiencing at least one acute respiratory infection.

1st Author (trial)	Year	Higher-dose vitamin D (%)	Lower-dose vitamin D (%)	Odds Ratio (95% CI)	% Weight
Grant	2014	47/76(61.8)	47/80(58.8)	1.14 (0.60, 2.16)	7.45
Tran	2014	84/200(42)	101/197(51.3)	0.69 (0.46, 1.02)	19.59
Martineau (ViDiFlu)***	2015	70/115(60.9)	45/79(57)	1.18 (0.66, 2.10)	9.08
Ginde	2016	17/55(30.9)	24/52(46.2)	0.52 (0.24, 1.15)	4.92
Aglipay	2017	184/349(52.7)	193/350(55.1)	0.91 (0.67, 1.22)	34.72
Hibbs	2018	92/153(60.1)	94/147(63.9)	0.85 (0.53, 1.36)	14.09
Lee	2018	31/31(100)	31/31(100)	1.00 (0.02, 51.98)	0.20
Rosendahl	2018	431/448(96.2)	440/449(98)	0.52 (0.23, 1.18)	4.58
Hauger	2019	21/39(53.8)	15/38(39.5)	1.79 (0.72, 4.42)	3.75
Reyes	2024	61/64(95.3)	64/69(92.8)	1.59 (0.36, 6.93)	1.42
NCT02404623 (Golan-Tripto)	Unpublished	14/14(100)	11/11(100)	1.26 (0.02, 68.54)	0.19
Overall (I-squared = 0.0%, p = 0.496)				0.87 (0.73, 1.04)	100.00

NOTE: Weights are from random effects analysis

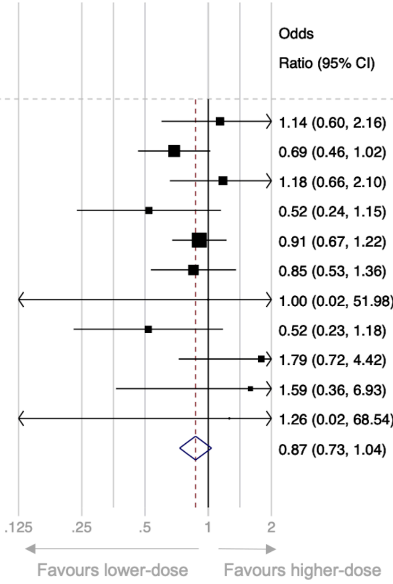

***This analysis includes data from the subset of ViDiFlu trial participants who were randomised to higher- vs. lower-dose vitamin D.

14 Appendix Figure 3: Forest plot of RCTs comparing effects of vitamin D vs. placebo, reporting proportion of participants experiencing at least one acute respiratory infection, by baseline 25-hydroxyvitamin D level. A) <25.0 nmol/L; B) 25.0 to 49.9 nmol/L; C) 50.0 to 74.9 nmol/L, and D) ≥75.0 nmol/L.

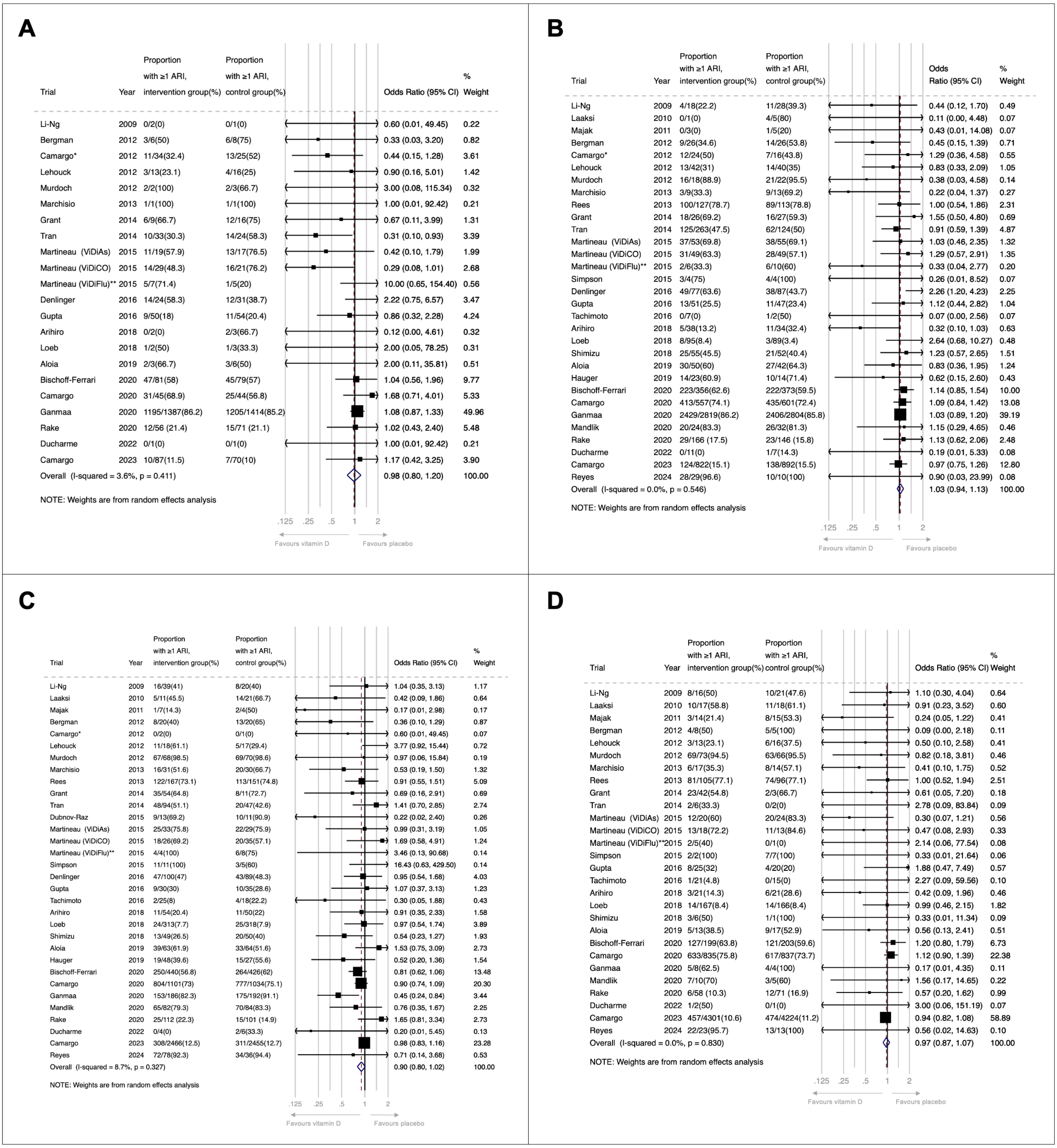

*Proportions for this trial were corrected for cluster randomisation using the calculated design effect of 4.12 for participants and clusters in the <25.0 nmol/L group (A) and the design effect of 1.23 for participants and clusters in the 25.0 to 49.9 nmol/L group (B). Design effect was incalculable for the 50.0 to 74.9 nmol/L group (C) due to the lack of power.
**This analysis includes data from the subset of ViDiFlu trial participants who were randomised to vitamin D vs. placebo control; correction for cluster randomisation was not possible due to the lack of power.

15 Appendix Figure 4: Forest plot of RCTs comparing effects of vitamin D vs. placebo, reporting proportion of participants experiencing at least one acute respiratory infection, by frequency of supplementation (daily vs. weekly vs. monthly to 3-monthly)

1st Author (trial)	Year	Proportion with ≥1 ARI, intervention group(%)	Proportion with ≥1 ARI, control group(%)	Odds Ratio (95% CI)	% Weight
**Daily dosing studies**					
Li-Ng	2009	32/81(39.5)	33/76(43.4)	0.85 (0.45, 1.61)	0.91
Laaksi	2010	39/80(48.8)	54/84(64.3)	0.53 (0.28, 0.99)	0.94
Urashima	2010	68/167(40.7)	69/167(41.3)	0.98 (0.63, 1.51)	1.82
Majak	2011	4/24(16.7)	11/24(45.8)	0.24 (0.06, 0.90)	0.22
Bergman	2012	26/62(41.9)	39/62(62.9)	0.43 (0.21, 0.88)	0.72
Camargo*	2012	13/40(32.5)	15/29(51.7)	0.45 (0.17, 1.20)	0.40
Marchisio	2013	26/58(44.8)	38/58(65.5)	0.43 (0.20, 0.90)	0.67
Rees	2013	303/399(75.9)	276/360(76.7)	0.96 (0.69, 1.34)	2.85
Grant	2014	94/157(59.9)	53/80(66.3)	0.76 (0.43, 1.33)	1.15
Urashima	2014	32/148(21.6)	17/99(17.2)	1.33 (0.69, 2.56)	0.87
Dubnov-Raz	2015	10/14(71.4)	10/11(90.9)	0.25 (0.02, 2.65)	0.07
Denlinger	2016	110/201(54.7)	93/207(44.9)	1.48 (1.00, 2.19)	2.21
Tachimoto	2016	4/54(7.4)	5/35(14.3)	0.48 (0.12, 1.93)	0.20
Shimizu	2018	41/110(37.3)	43/105(41)	0.86 (0.50, 1.48)	1.20
Aloia	2019	76/130(58.5)	72/130(55.4)	1.13 (0.69, 1.85)	1.47
Arihiro	2019	19/115(16.5)	30/108(27.8)	0.51 (0.27, 0.98)	0.88
Hauger	2019	36/77(46.8)	25/41(61)	0.56 (0.26, 1.21)	0.63
Bischoff-Ferrari	2020	647/1076(60.1)	652/1081(60.3)	0.99 (0.84, 1.18)	7.12
Mandlik	2020	92/116(79.3)	99/121(81.8)	0.85 (0.45, 1.62)	0.89
Huang	2022	1/135(0.7)	5/113(4.4)	0.16 (0.02, 1.40)	0.08
Camargo	2023	899/7676(11.7)	930/7641(12.2)	0.96 (0.87, 1.06)	11.23
Subtotal (I-squared = 44.8%, p = 0.014)				0.84 (0.73, 0.97)	36.55
.					
**Weekly dosing studies**					
Trilok-Kumar	2011	438/1034(42.4)	458/1030(44.5)	0.92 (0.77, 1.09)	7.04
Goodall	2014	70/258(27.1)	80/234(34.2)	0.72 (0.49, 1.05)	2.26
Simpson	2015	16/18(88.9)	14/16(87.5)	1.14 (0.14, 9.21)	0.09
Loeb	2018	50/577(8.7)	43/576(7.5)	1.18 (0.77, 1.80)	1.91
Ganmaa	2020	3783/4401(86)	3793/4418(85.9)	1.01 (0.89, 1.14)	9.86
Ducharme	2022	1/18(5.6)	3/15(20)	0.24 (0.02, 2.54)	0.07
Reyes	2024	125/133 (94)	59/61(96.7)	0.53 (0.11, 2.57)	0.16
Subtotal (I-squared = 0.0%, p = 0.435)				0.97 (0.88, 1.06)	21.39
.					
**Monthly to 3-monthly dosing studies**					
Manaseki-Holland	2010	97/224(43.3)	126/229(55)	0.62 (0.43, 0.90)	2.41
Lehouck	2012	30/86(34.9)	29/89(32.6)	1.11 (0.59, 2.07)	0.94
Manaseki-Holland	2012	260/1506(17.3)	245/1505(16.3)	1.07 (0.89, 1.30)	6.33
Murdoch	2012	154/161(95.7)	155/161(96.3)	0.85 (0.28, 2.59)	0.31
Tran	2014	185/397(46.6)	96/197(48.7)	0.92 (0.65, 1.29)	2.76
Martineau (ViDiAs)	2015	85/125(68)	93/125(74.4)	0.73 (0.42, 1.27)	1.20
Martineau (ViDiCO)	2015	76/122(62.3)	75/118(63.6)	0.95 (0.56, 1.60)	1.31
Martineau (ViDiFlu)**	2015	13/22(59.1)	13/24(54.2)	1.22 (0.38, 3.93)	0.28
Gupta	2016	39/156(25)	36/158(22.8)	1.13 (0.67, 1.90)	1.33
Camargo	2020	1882/2539(74.1)	1855/2517(73.7)	1.02 (0.90, 1.16)	9.53
Pham***	2020	5253/8000(65.7)	5310/8000(66.4)	0.97 (0.91, 1.03)	13.22
Rake	2020	73/395(18.5)	65/392 (16.6)	1.14 (0.79, 1.65)	2.44
Subtotal (I-squared = 0.0%, p = 0.565)				0.98 (0.93, 1.03)	42.06
.					
Overall (I-squared = 26.4%, p = 0.066)				0.94 (0.88, 1.00)	100.00

NOTE: Weights are from random effects analysis

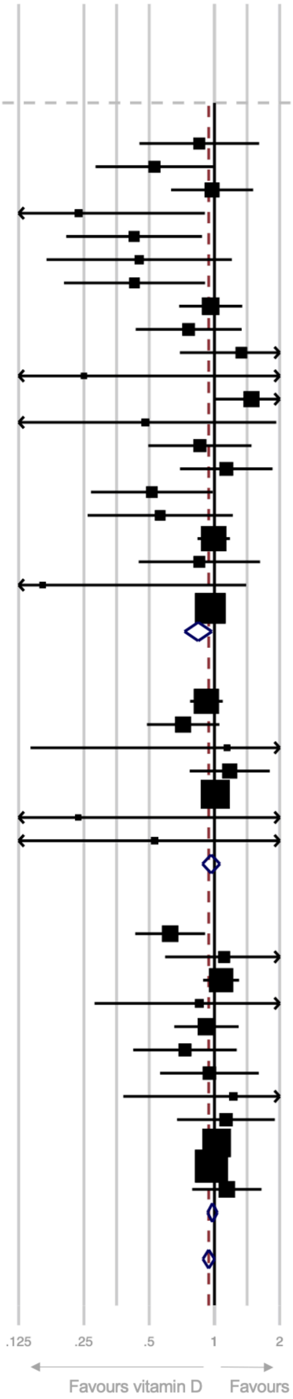

*Proportions for this trial were corrected for cluster randomisation using the calculated design effect of 3.49. **This analysis includes data from the subset of ViDiFlu trial participants who were randomised to vitamin D vs. placebo control; correction for cluster randomisation was not possible due to the lack of power. ***For this trial, participants were asked to report the occurrence of ARTI during the one month prior to completing each annual survey (max surveys=5). The numerator is the number of people who reported an ARTI on at least one survey. The ARTI outcomes for people who completed fewer than 5 surveys and who did not report an ARTI (N=2239; 14%) were estimated based on the % affected among those who completed all 5 surveys (N=12,152; 76%).

16 Appendix Figure 5: Forest plot of RCTs comparing effects of vitamin D vs. placebo, reporting proportion of participants experiencing at least one acute respiratory infection, by daily dose equivalents (<400 IU/day vs. 400-1000 IU/day vs. 1001-2000 IU/day vs. >2000 IU/day).

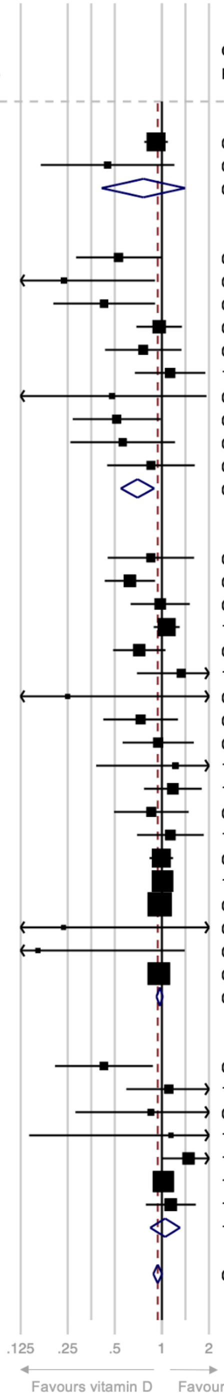

1st Author (trial)	Year	Proportion with ≥1 ARI, intervention group(%)	Proportion with ≥1 ARI, control group(%)	Odds Ratio (95% CI)	% Weight
Daily dose equivalent <400 IU					
Trilok-Kumar	2011	438/1034(42.4)	458/1030(44.5)	0.92 (0.77, 1.09)	7.25
Camargo*	2012	13/40(32.5)	15/29(51.7)	0.45 (0.17, 1.20)	0.43
Subtotal (I-squared = 49.0%, p = 0.161)				0.76 (0.41, 1.41)	7.68
.					
Daily dose equivalent 401-1000 IU					
Laaksi	2010	39/80(48.8)	54/84(64.3)	0.53 (0.28, 0.99)	1.01
Majak	2011	4/24(16.7)	11/24(45.8)	0.24 (0.06, 0.90)	0.23
Marchisio	2013	26/58(44.8)	38/58(65.5)	0.43 (0.20, 0.90)	0.72
Rees	2013	303/399(75.9)	276/360(76.7)	0.96 (0.69, 1.34)	3.03
Grant	2014	94/157(59.9)	53/80(66.3)	0.76 (0.43, 1.33)	1.23
Gupta	2016	39/156(25)	36/158(22.8)	1.13 (0.67, 1.90)	1.43
Tachimoto	2016	4/54(7.4)	5/35(14.3)	0.48 (0.12, 1.93)	0.22
Arihiro	2019	19/115(16.5)	30/108(27.8)	0.51 (0.27, 0.98)	0.95
Hauger	2019	36/77(46.8)	25/41(61)	0.56 (0.26, 1.21)	0.68
Mandlik	2020	92/116(79.3)	99/121(81.8)	0.85 (0.45, 1.62)	0.96
Subtotal (I-squared = 31.2%, p = 0.159)				0.70 (0.55, 0.89)	10.48
.					
Daily dose equivalent 1001-2000 IU					
Li-Ng	2009	32/81(39.5)	33/76(43.4)	0.85 (0.45, 1.61)	0.98
Manaseki-Holland	2010	97/224(43.3)	126/229(55)	0.62 (0.43, 0.90)	2.57
Urashima	2010	68/167(40.7)	69/167(41.3)	0.98 (0.63, 1.51)	1.95
Manaseki-Holland	2012	260/1506(17.3)	245/1505(16.3)	1.07 (0.89, 1.30)	6.55
Goodall	2014	70/258(27.1)	80/234(34.2)	0.72 (0.49, 1.05)	2.41
Urashima	2014	32/148(21.6)	17/99(17.2)	1.33 (0.69, 2.56)	0.94
Dubnov-Raz	2015	10/14(71.4)	10/11(90.9)	0.25 (0.02, 2.65)	0.08
Martineau (ViDiAs)	2015	85/125(68)	93/125(74.4)	0.73 (0.42, 1.27)	1.29
Martineau (ViDiCO)	2015	76/122(62.3)	75/118(63.6)	0.95 (0.56, 1.60)	1.41
Martineau (ViDiFlu)**	2015	13/22(59.1)	13/24(54.2)	1.22 (0.38, 3.93)	0.31
Loeb	2018	50/577(8.7)	43/576(7.5)	1.18 (0.77, 1.80)	2.04
Shimizu	2018	41/110(37.3)	43/105(41)	0.86 (0.50, 1.48)	1.29
Aloia	2019	76/130(58.5)	72/130(55.4)	1.13 (0.69, 1.85)	1.58
Bischoff-Ferrari	2020	647/1076(60.1)	652/1081(60.3)	0.99 (0.84, 1.18)	7.32
Ganmaa	2020	3783/4401(86)	3793/4418(85.9)	1.01 (0.89, 1.14)	9.94
Pham***	2020	5253/8000(65.7)	5310/8000(66.4)	0.97 (0.91, 1.03)	13.01
Ducharme	2022	1/18(5.6)	3/15(20)	0.24 (0.02, 2.54)	0.07
Huang	2022	1/135(0.7)	5/113(4.4)	0.16 (0.02, 1.40)	0.09
Camargo	2023	899/7676(11.7)	930/7641(12.2)	0.96 (0.87, 1.06)	11.21
Subtotal (I-squared = 1.6%, p = 0.437)				0.97 (0.92, 1.01)	65.03
.					
Daily dose equivalent >2000 IU					
Bergman	2012	26/62(41.9)	39/62(62.9)	0.43 (0.21, 0.88)	0.78
Lehouck	2012	30/86(34.9)	29/89(32.6)	1.11 (0.59, 2.07)	1.01
Murdoch	2012	154/161(95.7)	155/161(96.3)	0.85 (0.28, 2.59)	0.34
Simpson	2015	16/18(88.9)	14/16(87.5)	1.14 (0.14, 9.21)	0.10
Denlinger	2016	110/201(54.7)	93/207(44.9)	1.48 (1.00, 2.19)	2.36
Camargo	2020	1882/2539(74.1)	1855/2517(73.7)	1.02 (0.90, 1.16)	9.63
Rake	2020	73/395(18.5)	65/392 (16.6)	1.14 (0.79, 1.65)	2.60
Subtotal (I-squared = 37.1%, p = 0.146)				1.05 (0.84, 1.31)	16.81
.					
Overall (I-squared = 29.4%, p = 0.048)				0.94 (0.88, 1.00)	100.00

NOTE: Weights are from random effects analysis

*Proportions for this trial were corrected for cluster randomisation using the calculated design effect of 3.49. **This analysis includes data from the subset of ViDiFlu trial participants who were randomised to vitamin D vs. placebo control; correction for cluster randomisation was not possible due to the lack of power. ***For this trial, participants were asked to report the occurrence of ARTI during the one month prior to completing each annual survey (max surveys=5). The numerator is the number of people who reported an ARTI on at least one survey. The ARTI outcomes for people who completed fewer than 5 surveys and who did not report an ARTI (N=2239; 14%) were estimated based on the % affected among those who completed all 5 surveys (N=12,152; 76%).

17 Appendix Figure 6: Forest plot of RCTs comparing effects of vitamin D vs. placebo, reporting proportion of participants experiencing at least one acute respiratory infection, by trial duration (≤12 months vs. >12 months).

Trial	Year	Proportion with ≥1 ARI, intervention group(%)	Proportion with ≥1 ARI, control group(%)	Odds Ratio (95% CI)	% Weight
Studies with ≤12 months follow-up					
Li-Ng	2009	32/81(39.5)	33/76(43.4)	0.85 (0.45, 1.61)	0.91
Manaseki-Holland	2010	97/224(43.3)	126/229(55)	0.62 (0.43, 0.90)	2.41
Urashima	2010	68/167(40.7)	69/167(41.3)	0.98 (0.63, 1.51)	1.82
Camargo*	2012	13/40(32.5)	15/29(51.7)	0.45 (0.17, 1.20)	0.40
Goodall	2014	70/258(27.1)	80/234(34.2)	0.72 (0.49, 1.05)	2.26
Urashima	2014	32/148(21.6)	17/99(17.2)	1.33 (0.69, 2.56)	0.87
Dubnov-Raz	2015	10/14(71.4)	10/11(90.9)	0.25 (0.02, 2.65)	0.07
Simpson	2015	16/18(88.9)	14/16(87.5)	1.14 (0.14, 9.21)	0.09
Shimizu	2018	41/110(37.3)	43/105(41)	0.86 (0.50, 1.48)	1.20
Aloia	2019	76/130(58.5)	72/130(55.4)	1.13 (0.69, 1.85)	1.47
Hauger	2019	36/77(46.8)	25/41(61)	0.56 (0.26, 1.21)	0.63
Laaksi	2010	39/80(48.8)	54/84(64.3)	0.53 (0.28, 0.99)	0.94
Majak	2011	4/24(16.7)	11/24(45.8)	0.24 (0.06, 0.90)	0.22
Trilok-Kumar	2011	438/1034(42.4)	458/1030(44.5)	0.92 (0.77, 1.09)	7.04
Bergman	2012	26/62(41.9)	39/62(62.9)	0.43 (0.21, 0.88)	0.72
Lehouck	2012	30/86(34.9)	29/89(32.6)	1.11 (0.59, 2.07)	0.94
Marchisio	2013	26/58(44.8)	38/58(65.5)	0.43 (0.20, 0.90)	0.67
Grant	2014	94/157(59.9)	53/80(66.3)	0.76 (0.43, 1.33)	1.15
Tran	2014	185/397(46.6)	96/197(48.7)	0.92 (0.65, 1.29)	2.76
Martineau (ViDiAs)	2015	85/125(68)	93/125(74.4)	0.73 (0.42, 1.27)	1.20
Martineau (ViDiCO)	2015	76/122(62.3)	75/118(63.6)	0.95 (0.56, 1.60)	1.31
Martineau (ViDiFlu)**	2015	13/22(59.1)	13/24(54.2)	1.22 (0.38, 3.93)	0.28
Denlinger	2016	110/201(54.7)	93/207(44.9)	1.48 (1.00, 2.19)	2.21
Gupta	2016	39/156(25)	36/158(22.8)	1.13 (0.67, 1.90)	1.33
Tachimoto	2016	4/54(7.4)	5/35(14.3)	0.48 (0.12, 1.93)	0.20
Loeb	2018	50/577(8.7)	43/576(7.5)	1.18 (0.77, 1.80)	1.91
Arihiro	2019	19/115(16.5)	30/108(27.8)	0.51 (0.27, 0.98)	0.88
Mandlik	2020	92/116(79.3)	99/121(81.8)	0.85 (0.45, 1.62)	0.89
Ducharme	2022	1/18(5.6)	3/15(20)	0.24 (0.02, 2.54)	0.07
Huang	2022	1/135(0.7)	5/113(4.4)	0.16 (0.02, 1.40)	0.08
Camargo	2023	899/7676(11.7)	930/7641(12.2)	0.96 (0.87, 1.06)	11.23
Reyes	2024	125/133 (94)	59/61(96.7)	0.53 (0.11, 2.57)	0.16
Subtotal (I-squared = 32.7%, p = 0.040)				0.85 (0.76, 0.95)	48.33
.					
Studies with >12 months follow-up					
Manaseki-Holland	2012	260/1506(17.3)	245/1505(16.3)	1.07 (0.89, 1.30)	6.33
Murdoch	2012	154/161(95.7)	155/161(96.3)	0.85 (0.28, 2.59)	0.31
Rees	2013	303/399(75.9)	276/360(76.7)	0.96 (0.69, 1.34)	2.85
Camargo	2020	1882/2539(74.1)	1855/2517(73.7)	1.02 (0.90, 1.16)	9.53
Ganmaa	2020	3783/4401(86)	3793/4418(85.9)	1.01 (0.89, 1.14)	9.86
Rake	2020	73/395(18.5)	65/392 (16.6)	1.14 (0.79, 1.65)	2.44
Bischoff-Ferrari	2020	647/1076(60.1)	652/1081(60.3)	0.99 (0.84, 1.18)	7.12
Pham***	2020	5253/8000(65.7)	5310/8000(66.4)	0.97 (0.91, 1.03)	13.22
Subtotal (I-squared = 0.0%, p = 0.954)				0.99 (0.95, 1.04)	51.67
.					
Overall (I-squared = 26.4%, p = 0.066)				0.94 (0.88, 1.00)	100.00

NOTE: Weights are from random effects analysis

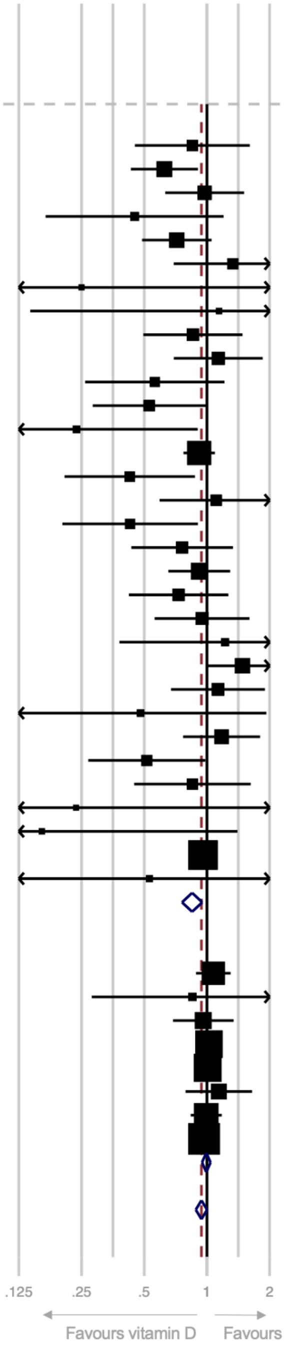

*Proportions for this trial were corrected for cluster randomisation using the calculated design effect of 3.49. **This analysis includes data from the subset of ViDiFlu trial participants who were randomised to vitamin D vs. placebo control; correction for cluster randomisation was not possible due to the lack of power. ***For this trial, participants were asked to report the occurrence of ARTI during the one month prior to completing each annual survey (max surveys=5). The numerator is the number of people who reported an ARTI on at least one survey. The ARTI outcomes for people who completed fewer than 5 surveys and who did not report an ARTI (N=2239; 14%) were estimated based on the % affected among those who completed all 5 surveys (N=12,152; 76%).

18 **Appendix Figure 7: Forest plot of RCTs comparing effects of vitamin D vs. placebo, reporting proportion of participants experiencing at least one acute respiratory infection, by the following age strata: A) <1 year; B) 1-15.99 years; C) 16-64.99 years, and D) ≥65 years**

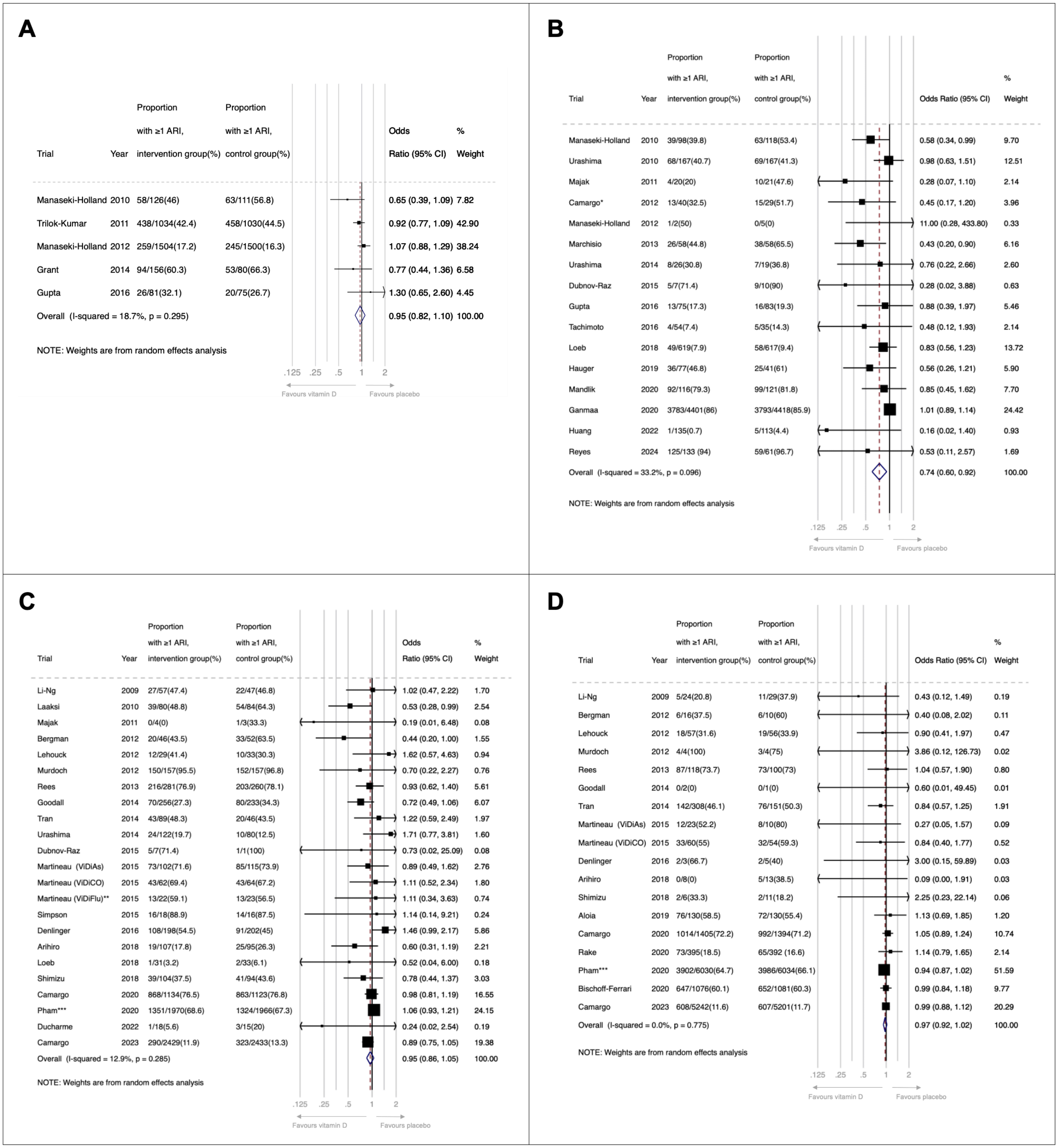

*Proportions for this trial were corrected for cluster randomisation using the calculated design effect of 3.49. **This analysis includes data from the subset of ViDiFlu trial participants who were randomised to vitamin D vs. placebo control. ***For this trial, participants were asked to report the occurrence of ARTI during the one month prior to completing each annual survey (max surveys=5). The numerator is the number of people who reported an ARTI on at least one survey. The ARTI outcomes for people who completed fewer than 5 surveys and who did not report an ARTI (N=2239; 14%) were estimated based on the % affected among those who completed all 5 surveys (N=12,152; 76%).

19 Appendix Figure 8: Forest plot of RCTs comparing effects of vitamin D vs. placebo, reporting proportion of participants experiencing at least one acute respiratory infection, by presence or absence of airway disease comorbidity.

1st Author (trial)	Year	Proportion with ≥1 ARI, intervention group(%)	Proportion with ≥1 ARI, control group(%)	Odds Ratio (95% CI)	% Weight
Asthma					
Majak	2011	4/24(16.7)	11/24(45.8)	0.24 (0.06, 0.90)	0.22
Martineau (ViDiAs)	2015	85/125(68)	93/125(74.4)	0.73 (0.42, 1.27)	1.20
Denlinger	2016	110/201(54.7)	93/207(44.9)	1.48 (1.00, 2.19)	2.21
Tachimoto	2016	4/54(7.4)	5/35(14.3)	0.48 (0.12, 1.93)	0.20
Subtotal (I-squared = 71.7%, p = 0.014)				0.73 (0.36, 1.49)	3.83
.					
COPD					
Lehouck	2012	30/86(34.9)	29/89(32.6)	1.11 (0.59, 2.07)	0.94
Martineau (ViDiCO)	2015	76/122(62.3)	75/118(63.6)	0.95 (0.56, 1.60)	1.31
Subtotal (I-squared = 0.0%, p = 0.706)				1.01 (0.68, 1.51)	2.25
.					
Other					
Li-Ng	2009	32/81(39.5)	33/76(43.4)	0.85 (0.45, 1.61)	0.91
Laaksi	2010	39/80(48.8)	54/84(64.3)	0.53 (0.28, 0.99)	0.94
Manaseki-Holland	2010	97/224(43.3)	126/229(55)	0.62 (0.43, 0.90)	2.41
Urashima	2010	68/167(40.7)	69/167(41.3)	0.98 (0.63, 1.51)	1.82
Trilok-Kumar	2011	438/1034(42.4)	458/1030(44.5)	0.92 (0.77, 1.09)	7.04
Bergman	2012	26/62(41.9)	39/62(62.9)	0.43 (0.21, 0.88)	0.72
Camargo*	2012	13/40(32.5)	15/29(51.7)	0.45 (0.17, 1.20)	0.40
Manaseki-Holland	2012	260/1506(17.3)	245/1505(16.3)	1.07 (0.89, 1.30)	6.33
Murdoch	2012	154/161(95.7)	155/161(96.3)	0.85 (0.28, 2.59)	0.31
Marchisio	2013	26/58(44.8)	38/58(65.5)	0.43 (0.20, 0.90)	0.67
Rees	2013	303/399(75.9)	276/360(76.7)	0.96 (0.69, 1.34)	2.85
Goodall	2014	70/258(27.1)	80/234(34.2)	0.72 (0.49, 1.05)	2.26
Grant	2014	94/157(59.9)	53/80(66.3)	0.76 (0.43, 1.33)	1.15
Tran	2014	185/397(46.6)	96/197(48.7)	0.92 (0.65, 1.29)	2.76
Urashima	2014	32/148(21.6)	17/99(17.2)	1.33 (0.69, 2.56)	0.87
Dubnov-Raz	2015	10/14(71.4)	10/11(90.9)	0.25 (0.02, 2.65)	0.07
Martineau (ViDiFlu)**	2015	13/22(59.1)	13/24(54.2)	1.22 (0.38, 3.93)	0.28
Simpson	2015	16/18(88.9)	14/16(87.5)	1.14 (0.14, 9.21)	0.09
Gupta	2016	39/156(25)	36/158(22.8)	1.13 (0.67, 1.90)	1.33
Loeb	2018	50/577(8.7)	43/576(7.5)	1.18 (0.77, 1.80)	1.91
Shimizu	2018	41/110(37.3)	43/105(41)	0.86 (0.50, 1.48)	1.20
Aloia	2019	76/130(58.5)	72/130(55.4)	1.13 (0.69, 1.85)	1.47
Arihiro	2019	19/115(16.5)	30/108(27.8)	0.51 (0.27, 0.98)	0.88
Hauger	2019	36/77(46.8)	25/41(61)	0.56 (0.26, 1.21)	0.63
Bischoff-Ferrari	2020	647/1076(60.1)	652/1081(60.3)	0.99 (0.84, 1.18)	7.12
Camargo	2020	1882/2539(74.1)	1855/2517(73.7)	1.02 (0.90, 1.16)	9.53
Ganmaa	2020	3783/4401(86)	3793/4418(85.9)	1.01 (0.89, 1.14)	9.86
Mandlik	2020	92/116(79.3)	99/121(81.8)	0.85 (0.45, 1.62)	0.89
Pham***	2020	5253/8000(65.7)	5310/8000(66.4)	0.97 (0.91, 1.03)	13.22
Rake	2020	73/395(18.5)	65/392 (16.6)	1.14 (0.79, 1.65)	2.44
Ducharme	2022	1/18(5.6)	3/15(20)	0.24 (0.02, 2.54)	0.07
Huang	2022	1/135(0.7)	5/113(4.4)	0.16 (0.02, 1.40)	0.08
Camargo	2023	899/7676(11.7)	930/7641(12.2)	0.96 (0.87, 1.06)	11.23
Reyes	2024	125/133 (94)	59/61(96.7)	0.53 (0.11, 2.57)	0.16
Subtotal (I-squared = 21.4%, p = 0.136)				0.94 (0.89, 1.00)	93.93
.					
Overall (I-squared = 26.4%, p = 0.066)				0.94 (0.88, 1.00)	100.00

NOTE: Weights are from random effects analysis

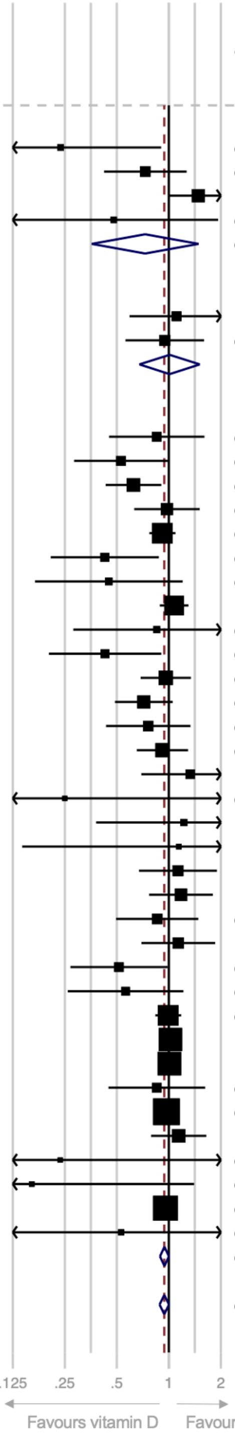

*Proportions for this trial were corrected for cluster randomisation using the calculated design effect of 3.49. **This analysis includes data from the subset of ViDiFlu trial participants who were randomised to vitamin D vs. placebo control; correction for cluster randomisation was not possible due to the lack of power. ***For this trial, participants were asked to report the occurrence of ARTI during the one month prior to completing each annual survey (max surveys=5). The numerator is the number of people who reported an ARTI on at least one survey. The ARTI outcomes for people who completed fewer than 5 surveys and who did not report an ARTI (N=2239; 14%) were estimated based on the % affected among those who completed all 5 surveys (N=12,152; 76%).

Appendix Figure 9: Funnel plot of placebo-controlled RCTs reporting proportion of participants experiencing 1 or more acute respiratory infection.

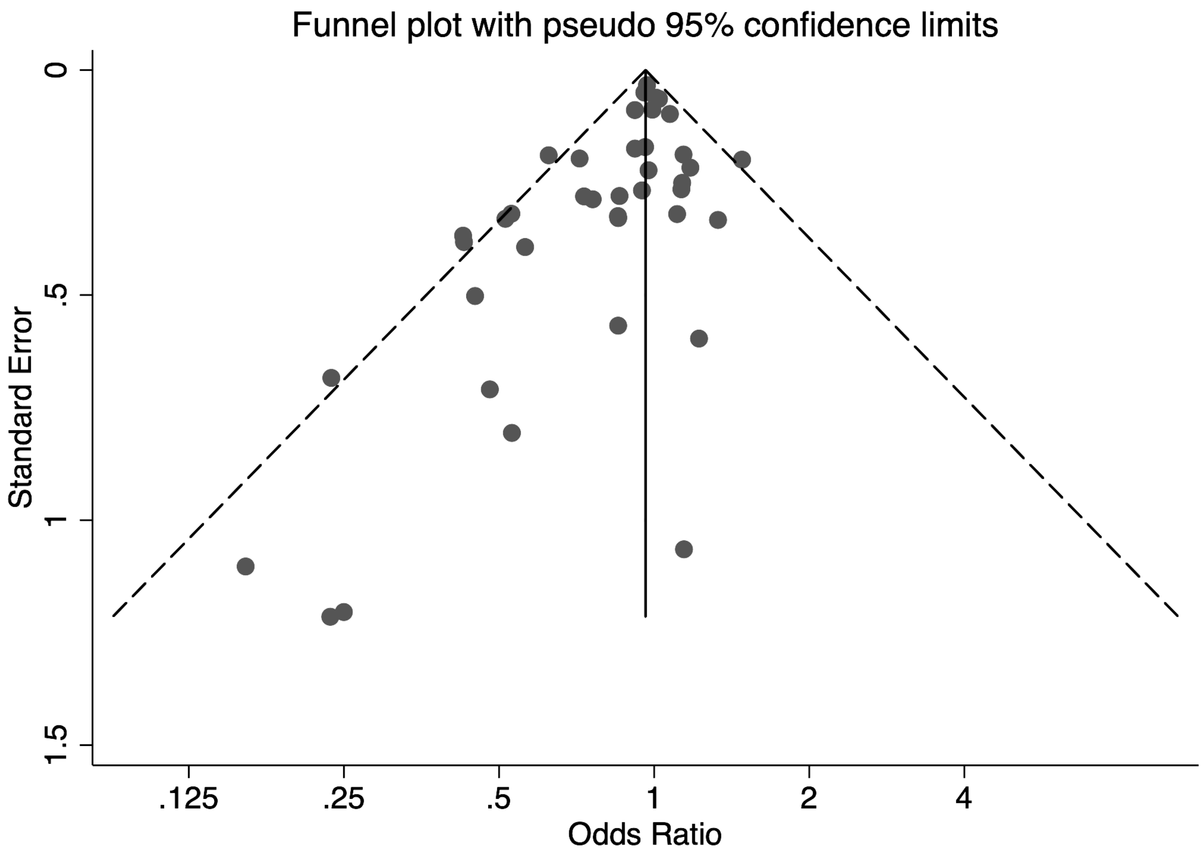

Egger’s test for publication bias: P=0.002

21 References

1. Martineau AR, Jolliffe DA, Hooper RL, et al. Vitamin D supplementation to prevent acute respiratory tract infections: systematic review and meta-analysis of individual participant data. *BMJ* 2017; **356**: i6583.
2. Camargo CA, Jr., Ganmaa D, Frazier AL, et al. Randomized trial of vitamin D supplementation and risk of acute respiratory infection in Mongolia. *Pediatrics* 2012; **130**(3): e561-7.
3. Murdoch DR, Slow S, Chambers ST, et al. Effect of vitamin D3 supplementation on upper respiratory tract infections in healthy adults: the VIDARIS randomized controlled trial. *JAMA* 2012; **308**(13): 1333-9.
4. Rees JR, Hendricks K, Barry EL, et al. Vitamin D3 Supplementation and Upper Respiratory Tract Infections in a Randomized, Controlled Trial. *Clin Infect Dis* 2013.
5. Tachimoto H, Mezawa H, Segawa T, Akiyama N, Ida H, Urashima M. Improved Control of Childhood Asthma with Low-Dose, Short-Term Vitamin D Supplementation: A Randomized, Double-Blind, Placebo-Controlled Trial. *Allergy* 2016.
6. Tran B, Armstrong BK, Ebeling PR, et al. Effect of vitamin D supplementation on antibiotic use: a randomized controlled trial. *Am J Clin Nutr* 2014; **99**(1): 156-61.
7. Urashima M, Mezawa H, Noya M, Camargo CA, Jr. Effects of vitamin D supplements on influenza A illness during the 2009 H1N1 pandemic: a randomized controlled trial. *Food & function* 2014; **5**(9): 2365-70.
8. Urashima M, Segawa T, Okazaki M, Kurihara M, Wada Y, Ida H. Randomized trial of vitamin D supplementation to prevent seasonal influenza A in schoolchildren. *The American journal of clinical nutrition* 2010; **91**(5): 1255-60.
9. Bakbergenuly I, Hoaglin DC, Kulinskaya E. Pitfalls of using the risk ratio in meta-analysis. *Res Synth Methods* 2019; **10**(3): 398-419.
10. Higgins JPT, Thomas J, Chandler J, et al. Cochrane Handbook for Systematic Reviews of Interventions version 6.2 (updated February 2021): Cochrane Available from www.training.cochrane.org/handbook. 2021.
11. Jolliffe DA, Camargo CA, Jr., Sluyter JD, et al. Vitamin D supplementation to prevent acute respiratory infections: a systematic review and meta-analysis of aggregate data from randomised controlled trials. *Lancet Diabetes Endocrinol* 2021; **9**(5): 276-92.
12. jolliffe DA, greenberg L, hooper RL, et al. Vitamin D supplementation to prevent asthma exacerbations: systematic review and meta-analysis of individual participant data. *Lancet Respiratory Medicine*.
13. Jolliffe DA, Greenberg L, Hooper RL, et al. Vitamin D to prevent exacerbations of COPD: systematic review and meta-analysis of individual participant data from randomised controlled trials. *Thorax* 2019; **74**(4): 337-45.
14. Reyes ML, Vizcaya C, Borzutzky A, et al. Study of Vitamin D for the Prevention of Acute Respiratory Infections in Children (NCT02046577). https://clinicaltrials.gov/ct2/show/NCT02046577.
15. Majak P, Olszowiec-Chlebna M, Smejda K, Stelmach I. Vitamin D supplementation in children may prevent asthma exacerbation triggered by acute respiratory infection. *J Allergy Clin Immunol* 2011; **127**(5): 1294-6.
16. Dubnov-Raz G, Rinat B, Hemila H, Choleva L, Cohen AH, Constantini NW. Vitamin D supplementation and upper respiratory tract infections in adolescent swimmers: a randomized controlled trial. *Pediatric exercise science* 2015; **27**(1): 113-9.
17. Loeb M, Dang AD, Thiem VD, et al. Effect of Vitamin D supplementation to reduce respiratory infections in children and adolescents in Vietnam: A randomized controlled trial. *Influenza Other Respir Viruses* 2019; **13**(2): 176-83.
18. Somnath SH, Biswal N, Chandrasekaran V, Jagadisan B, Bobby Z. Therapeutic effect of vitamin D in acute lower respiratory infection: A randomized controlled trial. *Clin Nutr ESPEN* 2017; **20**: 24-8.

19. Jung HC, Seo M-W, Lee S, Kim SW, Song JK. Vitamin D_3 Supplementation Reduces the Symptoms of Upper Respiratory Tract Infection during Winter Training in Vitamin D-Insufficient Taekwondo Athletes: A Randomized Controlled Trial. *Int J Environ Res Public Health* 2018; **15**(9): 2003.
20. Ramos-Martinez E, Lopez-Vancell MR, Fernandez de Cordova-Aguirre JC, et al. Reduction of respiratory infections in asthma patients supplemented with vitamin D is related to increased serum IL-10 and IFNgamma levels and cathelicidin expression. *Cytokine* 2018; **108**: 239-46.
21. Zhou J, Du J, Huang L, Wang Y, Shi Y, Lin H. Preventive Effects of Vitamin D on Seasonal Influenza A in Infants: A Multicenter, Randomized, Open, Controlled Clinical Trial. *The Pediatric infectious disease journal* 2018; **37**(8): 749-54.
22. Hueniken K, Aglipay M, Birken CS, et al. Effect of High-Dose Vitamin D Supplementation on Upper Respiratory Tract Infection Symptom Severity in Healthy Children. *Pediatr Infect Dis J* 2019; **38**(6): 564-8.
23. Aglipay M, Birken CS, Parkin PC, et al. Effect of High-Dose vs Standard-Dose Wintertime Vitamin D Supplementation on Viral Upper Respiratory Tract Infections in Young Healthy Children. *JAMA* 2017; **318**(3): 245-54.
24. Singh N, Kamble D, Mahantshetti NS. Effect of Vitamin D Supplementation in the Prevention of Recurrent Pneumonia in Under-Five Children. *Indian journal of pediatrics* 2019; **86**(12): 1105-11.
25. Jolliffe DA, Holt H, Greenig M, et al. Vitamin D Supplements for Prevention of Covid-19 or other Acute Respiratory Infections: a Phase 3 Randomized Controlled Trial (CORONAVIT). *BMJ* 2022; **378**: e071230.
26. van Helmond N, Brobyn TL, LaRiccia PJ, et al. Vitamin D3 Supplementation at 5000 IU Daily for the Prevention of Influenza-like Illness in Healthcare Workers: A Pragmatic Randomized Clinical Trial. *Nutrients* 2022; **15**(1).
27. Villasis-Keever MA, Lopez-Alarcon MG, Miranda-Novales G, et al. Efficacy and Safety of Vitamin D Supplementation to Prevent COVID-19 in Frontline Healthcare Workers. A Randomized Clinical Trial. *Arch Med Res* 2022; **53**(4): 423-30.
28. Li-Ng M, Aloia JF, Pollack S, et al. A randomized controlled trial of vitamin D3 supplementation for the prevention of symptomatic upper respiratory tract infections. *Epidemiol Infect* 2009; **137**(10): 1396-404.
29. Manaseki-Holland S, Qader G, Isaq Masher M, et al. Effects of vitamin D supplementation to children diagnosed with pneumonia in Kabul: a randomised controlled trial. *Trop Med Int Health* 2010; **15**(10): 1148-55.
30. Laaksi I, Ruohola JP, Mattila V, Auvinen A, Ylikomi T, Pihlajamaki H. Vitamin D supplementation for the prevention of acute respiratory tract infection: a randomized, double-blinded trial among young Finnish men. *J Infect Dis* 2010; **202**(5): 809-14.
31. Kumar GT, Sachdev HS, Chellani H, et al. Effect of weekly vitamin D supplements on mortality, morbidity, and growth of low birthweight term infants in India up to age 6 months: randomised controlled trial. *BMJ* 2011; **342**: d2975.
32. Lehouck A, Mathieu C, Carremans C, et al. High doses of vitamin D to reduce exacerbations in chronic obstructive pulmonary disease: a randomized trial. *Ann Intern Med* 2012; **156**(2): 105-14.
33. Manaseki-Holland S, Maroof Z, Bruce J, et al. Effect on the incidence of pneumonia of vitamin D supplementation by quarterly bolus dose to infants in Kabul: a randomised controlled superiority trial. *Lancet* 2012; **379**(9824): 1419-27.
34. Bergman P, Norlin AC, Hansen S, et al. Vitamin D3 supplementation in patients with frequent respiratory tract infections: a randomised and double-blind intervention study. *BMJ Open* 2012; **2**(6): e001663.
35. Marchisio P, Consonni D, Baggi E, et al. Vitamin D supplementation reduces the risk of acute otitis media in otitis-prone children. *Pediatr Infect Dis J* 2013; **32**(10): 1055-60.

36. Goodall EC, Granados AC, Luinstra K, et al. Vitamin D3 and gargling for the prevention of upper respiratory tract infections: a randomized controlled trial. *BMC infectious diseases* 2014; **14**: 273.
37. Grant CC, Kaur S, Waymouth E, et al. Reduced primary care respiratory infection visits following pregnancy and infancy vitamin D supplementation: a randomised controlled trial. *Acta Paediatr* 2014.
38. Martineau AR, James WY, Hooper RL, et al. Vitamin D3 supplementation in patients with chronic obstructive pulmonary disease (ViDiCO): a multicentre, double-blind, randomised controlled trial. *The lancet Respiratory medicine* 2015; **3**(2): 120-30.
39. Martineau AR, MacLaughlin BD, Hooper RL, et al. Double-blind randomised placebo-controlled trial of bolus-dose vitamin D3 supplementation in adults with asthma (ViDiAs). *Thorax* 2015; **70**(5): 451-7.
40. Martineau AR, Hanifa Y, Witt KD, et al. Double-blind randomised controlled trial of vitamin D3 supplementation for the prevention of acute respiratory infection in older adults and their carers (ViDiFlu). *Thorax* 2015.
41. Simpson SJ, van der Mei I, Stewart N, Blizzard L, Tettey P, Taylor B. Weekly cholecalciferol supplementation results in significant reductions in infection risk among the vitamin D deficient: results from the CIPRIS pilot RCT. *BMC Nutrition* 2015; **1**(7).
42. Denlinger LC, King TS, Cardet JC, et al. Vitamin D Supplementation and the Risk of Colds in Patients with Asthma. *Am J Respir Crit Care Med* 2016; **193**(6): 634-41.
43. Ginde AA, Blatchford P, Breese K, et al. High-Dose Monthly Vitamin D for Prevention of Acute Respiratory Infection in Older Long-Term Care Residents: A Randomized Clinical Trial. *J Am Geriatr Soc* 2017; **65**(3): 496-503.
44. Gupta P, Dewan P, Shah D, et al. Vitamin D Supplementation for Treatment and Prevention of Pneumonia in Under-five Children: A Randomized Double-blind Placebo Controlled Trial. *Indian Pediatr* 2016; **53**(11): 967-76.
45. Arihiro S, Nakashima A, Matsuoka M, et al. Randomized Trial of Vitamin D Supplementation to Prevent Seasonal Influenza and Upper Respiratory Infection in Patients With Inflammatory Bowel Disease. *Inflamm Bowel Dis* 2019; **25**(6): 1088-95.
46. Hibbs AM, Ross K, Kerns LA, et al. Effect of Vitamin D Supplementation on Recurrent Wheezing in Black Infants Who Were Born Preterm: The D-Wheeze Randomized Clinical Trial. *JAMA* 2018; **319**(20): 2086-94.
47. Lee MT, Kattan M, Fennoy I, et al. Randomized phase 2 trial of monthly vitamin D to prevent respiratory complications in children with sickle cell disease. *Blood Adv* 2018; **2**(9): 969-78.
48. Rosendahl J, Valkama S, Holmlund-Suila E, et al. Effect of Higher vs Standard Dosage of Vitamin D3 Supplementation on Bone Strength and Infection in Healthy Infants: A Randomized Clinical Trial. *JAMA pediatrics* 2018; **172**(7): 646-54.
49. Shimizu Y, Ito Y, Yui K, Egawa K, Orimo H. Intake of 25-Hydroxyvitamin D3 Reduces Duration and Severity of Upper Respiratory Tract Infection: A Randomized, Double-Blind, Placebo-Controlled, Parallel Group Comparison Study. *J Nutr Health Aging* 2018; **22**(4): 491-500.
50. Aloia JF, Islam S, Mikhail M. Vitamin D and Acute Respiratory Infections-The PODA Trial. *Open forum infectious diseases* 2019; **6**(9): ofz228.
51. Hauger H, Ritz C, Mortensen C, et al. Winter cholecalciferol supplementation at 55°N has little effect on markers of innate immune defense in healthy children aged 4–8 years: a secondary analysis from a randomized controlled trial. *European Journal of Nutrition* 2019; **58**(4): 1453-62.
52. Bischoff-Ferrari HA, Vellas B, Rizzoli R, et al. Effect of Vitamin D Supplementation, Omega-3 Fatty Acid Supplementation, or a Strength-Training Exercise Program on Clinical Outcomes in Older Adults: The DO-HEALTH Randomized Clinical Trial. *JAMA* 2020; **324**(18): 1855-68.

53. Camargo CA, Sluyter J, Stewart AW, et al. Effect of monthly high-dose vitamin D supplementation on acute respiratory infections in older adults: A randomized controlled trial. *Clin Infect Dis* 2020; **71**: 311-7.
54. Ganmaa D, Uyanga B, Zhou X, et al. Vitamin D Supplements and Prevention of Tuberculosis Infection and Disease. *N Engl J Med* 2020; **383**: 359-68.
55. Mandlik R, Mughal Z, Khadilkar A, et al. Occurrence of infections in schoolchildren subsequent to supplementation with vitamin D-calcium or zinc: a randomized, double-blind, placebo-controlled trial. *Nutr Res Pract* 2020; **14**(2): 117-26.
56. Pham H, Waterhouse M, Baxter C, et al. The effect of vitamin D supplementation on acute respiratory tract infection in older Australian adults: an analysis of data from the D-Health Trial: a randomised controlled trial. *The lancet Diabetes & endocrinology* 2020; **in press**.
57. Rake C, Gilham C, Bukasa L, et al. High-dose oral vitamin D supplementation and mortality in people aged 65-84 years: the VIDAL cluster feasibility RCT of open versus double-blind individual randomisation. *Health Technol Assess* 2020; **24**(10): 1-54.
58. Ducharme FM, Tremblay C, Golchi S, et al. Prevention of COVID-19 with oral vitamin D supplemental therapy in essential healthcare teams (PROTECT): protocol for a multicentre, triple-blind, randomised, placebo-controlled trial. *BMJ Open* 2023; **13**(5): e064058.
59. Huang YN, Chi H, Chiu NC, et al. A randomized trial of vitamin D supplementation to prevent seasonal influenza and enterovirus infection in children. *J Microbiol Immunol Infect* 2022; **55**(5): 803-11.
60. Camargo CA, Jr., Schaumberg DA, Friedenberg G, et al. Effect of Daily Vitamin D Supplementation on Risk of Upper Respiratory Infection in Older Adults: A Randomized Controlled Trial. *Clinical infectious diseases : an official publication of the Infectious Diseases Society of America* 2024; **78**(5): 1162-9.
61. Reyes ML, Vizcaya C, Le Roy C, et al. Weekly Vitamin D Supplementation to Prevent Acute Respiratory Infections in Young Children at Different Latitudes: A Randomized Controlled Trial. *J Pediatr* 2024; **275**: 114249.
62. Golan-Tripto I. The Effect of Vitamin D Administration to Premature Infants on Vitamin D Status and Respiratory Morbidity (NCT02404623). https://clinicaltrials.gov/ct2/show/NCT02404623.
